# The Immunological Biography of the Carotid Plaque: From Trained Immunity and Clonal Lymphocytes to Spatial Immune Niches and Plaque Vulnerability

**DOI:** 10.3390/cells15211995

**Published:** 2026-10-31

**Authors:** Zbigniew Krasiński, Giuseppe Maria Raffa, Mariusz Kowalewski, Beata Krasińska, Calogera Pisano, Stefano Cannata, Piotr Suwalski, Tomasz Urbanowicz

**Affiliations:** 1 Department of Vascular, Endovascular Surgery, Angiology and Phlebology, Poznan University of Medical Science, ½ Dluga Street, 61-848 Poznań, Poland; 2 Department of Precision Medicine in Medical Surgical and Critical Area (Me.Pre.C.C.), University of Palermo, 90134 Palermo, Italy; 3 Department of Research, IRCCS ISMETT, Via Ernesto Tricomi, 5, 90127 Palermo, Italy; 4 Department of Cardiac Surgery, Center of Postgraduate Medical Education, Central Clinical Hospital of the Ministry of Interior, 02-507 Warszawa, Poland; 5 Thoracic Research Center, Collegium Medicum, Nicolaus Copernicus University, 85-090 Bydgoszcz, Poland; 6 Department of Hypertensiology, Angiology, and Internal Medicine, Poznan University of Medical Sciences, ½ Dluga Street, 61-848 Poznań, Poland; 7 Cardiac Surgery and Transplantology Department, Poznan University of Medical Sciences, ½ Długa Street, 61-848 Poznań, Poland

**Keywords:** carotid atherosclerosis, carotid plaque, trained immunity, adaptive immunity, immune memory, T-cell clonality, spatial transcriptomics, single-cell RNA sequencing, plaque vulnerability, vascular inflammation

## Abstract

**Highlights:**

**What are the main findings?**

- The immune state of the carotid plaque reflects a cumulative “immunological biography” shaped by systemic exposures, trained innate immunity, adaptive immune memory, clonal lymphocyte selection, and local tissue remodeling.
- Single-cell, immune-repertoire, and spatial approaches reveal that plaque biology depends not only on which immune cells are present, but also on their functional state, clonal history, anatomical localization, and cellular interactions.

**What are the implications of the main findings?**

- Integrating circulating immune profiles with plaque tissue, TCR/BCR clonality, and spatially resolved molecular data may provide a more biologically informative characterization of carotid disease than stenosis severity or isolated inflammatory biomarkers alone.
- The proposed Carotid Plaque Immune Phenotype (CPIP) provides a conceptual framework for translating multidimensional immune information into testable plaque phenotypes and for developing future studies of plaque vulnerability and cerebrovascular risk.

**Abstract:**

Atherosclerotic carotid plaques are shaped by years of interaction between systemic risk factors, hematopoietic conditioning, vascular injury, and local immune responses, yet conventional assessment captures only a limited snapshot of this biological history. This review introduces the concept of the “immunological biography” of the carotid plaque and examines how trained innate immunity, macrophage state and defective resolution, adaptive immune memory, T- and B-cell clonal selection, and spatially organized immune niches contribute to plaque heterogeneity and vulnerability. The literature was identified through a structured PubMed search complemented by reference screening, with emphasis on human carotid tissue, paired blood–plaque studies, immune-repertoire analyses, and single-cell and spatial technologies. Current evidence indicates that immune-cell abundance alone incompletely characterizes plaque biology: functional state, antigen experience, clonality, tissue localization, and interactions with vascular and stromal cells provide additional levels of biological information. We propose the Carotid Plaque Immune Phenotype (CPIP) as an integrative framework linking systemic immune history with cellular state, clonal architecture, spatial organization, and clinically relevant plaque features. Combining circulating biomarkers with tissue-resolved immune profiling may ultimately improve biological phenotyping of carotid atherosclerosis and provide a framework for prospective studies linking plaque immune architecture with cerebrovascular events.

## 1. The Immune Origins of the Carotid Plaque: From Endothelial Activation to Trained Immunity

The inflammatory environment of a carotid plaque is not established when the lesion becomes clinically apparent. Long before a patient develops significant stenosis, transient ischemic symptoms, or stroke, the arterial wall has been exposed to years of mechanical stress, lipid accumulation, metabolic disturbance, and systemic inflammation [1,2]. The immune cells subsequently recovered from an advanced plaque enter a tissue with a history, and many of these cells themselves arrive with a history acquired in circulation or during hematopoiesis [3,4]. This is an important point to consider when explaining why plaques of apparently similar size and morphology can behave very differently [5].

**Figure 1:**
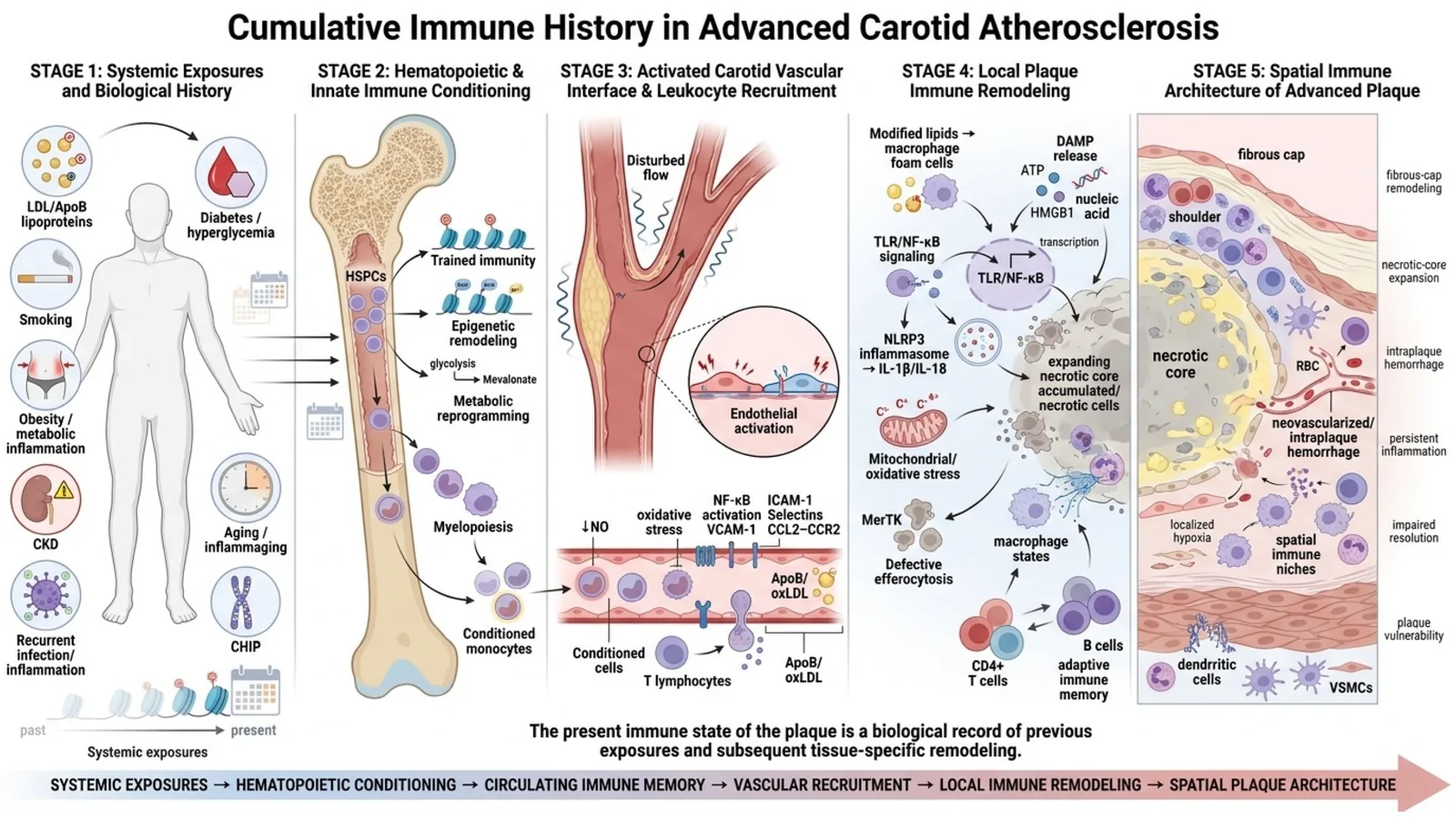
The Immunological Biography of the Carotid Plaque: From Systemic Exposure to Local Immune Architecture. **Abbreviations:** ApoB, apolipoprotein B; ATP, adenosine triphosphate; CCL2, C-C motif chemokine ligand 2; CCR2, C-C motif chemokine receptor 2; CD4, cluster of differentiation 4; CHIP, clonal hematopoiesis of indeterminate potential; CKD, chronic kidney disease; DAMP, damage-associated molecular pattern; HMGB1, high-mobility group box 1; HSPCs, hematopoietic stem and progenitor cells; ICAM-1, intercellular adhesion molecule 1; IL, interleukin; LDL, low-density lipoprotein; MerTK, MER proto-oncogene tyrosine kinase; NF-κB, nuclear factor kappa B; NLRP3, NOD-, LRR- and pyrin domain-containing protein 3; NO, nitric oxide; oxLDL, oxidized low-density lipoprotein; RBC, red blood cell; TLR, Toll-like receptor; VCAM-1, vascular cell adhesion molecule 1; VSMCs, vascular smooth muscle cells. Created by Urbanowicz T, on 3 October 2026 with www.figurelabs.ai (ID: FL-PUB-20261003-NPXHOQ).

Hypercholesterolemia, hypertension, diabetes, smoking, aging, chronic kidney disease, infections, and systemic inflammatory states leave partly overlapping but not necessarily equivalent effects on these compartments [6,7,8]. Previous ones condition the inflammatory response to a new stimulus [9,10]. In this sense, advanced carotid atherosclerosis is not simply chronic inflammation maintained over time; it is inflammation occurring in tissue and immune cells that have been repeatedly remodeled by earlier events [11,12]. These observations place the advanced carotid plaque within a biological continuum rather than at the endpoint of an isolated inflammatory process. Systemic exposures, hematopoietic conditioning, and local vascular signals accumulate over time and ultimately converge within the arterial wall [13,14]. This concept of an immunological biography, along with the principal biological levels through which it develops, is summarized in Figure 1.

The carotid bifurcation provides a particularly instructive setting in which this process begins. Disturbed and oscillatory flow modifies endothelial mechanotransduction and favors a phenotype that is more permissive to inflammation and leukocyte recruitment [15,16]. This hemodynamic susceptibility becomes biologically important when combined with systemic risk factors. Reduced nitric oxide bioavailability, oxidative stress, and activation of inflammatory transcriptional programs increase endothelial expression of VCAM-1, ICAM-1, selectins, and chemokines, including those involved in CCL2–CCR2-dependent monocyte recruitment [17,18]. Consequently, the endothelium changes from a surface that limits cellular adhesion to one that actively participates in the selection and recruitment of circulating leukocytes [19]. Endothelial cells remain active participants throughout plaque development [20]. They continuously encounter altered shear stress, circulating lipoproteins, inflammatory mediators, metabolic abnormalities, and signals released from the plaque [21]. They are both targets of systemic injury and regulators of local immunity [22,23].

Retention of ApoB-containing lipoproteins in the intima changes the nature of this inflammatory environment [24]. Once retained, lipoproteins undergo oxidation, aggregation, and other modifications and can no longer be regarded merely as accumulated lipid [25]. Modified lipoproteins interact with scavenger receptors and innate immune pathways, influencing the phenotype of macrophages and other vascular cells [26]. At the same time, cells exposed to lipid overload, oxidative stress, mitochondrial dysfunction, and mechanical injury release endogenous danger signals [27]. Extracellular ATP, HMGB1, nucleic acids, mitochondrial components, cholesterol crystals, and material released from necrotic cells contribute to an expanding pool of damage-associated molecular patterns (DAMPs) [28].

Lipid accumulation generates cellular stress; cellular stress generates inflammatory signals; inflammation modifies lipid handling and cell survival [29]. Pattern-recognition receptors help translate these signals into inflammatory responses [30]. Toll-like receptor pathways provide one important route, particularly through transcriptional programs involving NF-κB and related signaling networks [31]. The NLRP3 inflammasome is another major signaling platform that integrates multiple forms of metabolic and cellular stress into a coordinated inflammatory response [32]. Cholesterol crystals, lysosomal injury, mitochondrial dysfunction, oxidative stress, and ionic disturbances can contribute to NLRP3 activation, with subsequent caspase-1-dependent processing of IL-1β and IL-18 [33]. The importance of these pathways is well established, but their representation as a simple sequence—lipid accumulation, TLR activation, NLRP3 signaling, cytokine release—probably understates the biology of the human plaque [34]. These processes occur in parallel and influence one another [35]. Mitochondrial damage changes cellular metabolism and redox signaling [36]. Defective clearance of apoptotic cells increases the availability of endogenous danger signals [37]. Cytokines modulate endothelial activation and monocyte recruitment, while newly recruited cells serve as additional sources of inflammatory mediators [38]. The inflammatory state of the plaque consequently emerges from interacting circuits rather than from a single dominant pathway [39].

Monocytes, after adhesion and transendothelial migration, encounter an environment rich in modified lipids, cytokines, extracellular matrix, apoptotic cells, and metabolic stress [40,41]. Following recruitment, monocytes differentiate and adapt to the plaque microenvironment, giving rise to a spectrum of macrophage states that extends well beyond the traditional M1/M2 classification [42]. Human and experimental single-cell studies have revealed inflammatory, lipid-associated, interferon-responsive, proliferative, and resident-like macrophage populations, together with transitional states that resist simple classification [43]. Macrophage identity within the plaque is not fixed at recruitment and continually reshaped by the local environment [44,45].

Lipid uptake is an important determinant of macrophage phenotype, although it is only one component of the local environment [46]. Hypoxia, mitochondrial stress, iron availability, cytokine exposure, cell–cell interactions, and the efficiency of efferocytosis also influence macrophage function and adaptation within the plaque [47,48]. Efferocytosis is particularly relevant in advanced lesions. Efficient removal of apoptotic cells can limit secondary inflammation, whereas failed clearance permits cellular debris to accumulate and promotes expansion of the necrotic core [49]. A macrophage that initially entered the plaque to remove modified lipid and dying cells may become part of an environment in which the capacity for homeostatic clearance is progressively exceeded [50]. The transition from recruitment to dysfunction is not abrupt, and it is unlikely to occur uniformly throughout the plaque [51].

An equally important development in atherosclerosis immunology has been the recognition that monocytes arriving at the arterial wall are not necessarily immunologically equivalent. Their functional state can be influenced before entering the lesion [52]. Hypercholesterolemia and chronic inflammatory signaling affect hematopoietic stem and progenitor cells, promoting myelopoiesis and increasing the production of monocytes and other myeloid populations available for recruitment [53]. The bone marrow is more than a passive reservoir supplying cells to an inflamed artery. It participates in the disease process by determining both the quantity and, potentially, the functional characteristics of the cells entering the circulation [54]. The concept challenged the traditional assumption that immunological memory is restricted to antigen-specific lymphocytes [11]. Following particular inflammatory or metabolic exposures, innate immune cells can acquire a modified response to later stimulation. Because circulating monocytes are relatively short-lived, the persistence of this phenomenon is especially interesting when reprogramming occurs at the level of hematopoietic progenitors. Modified lipoproteins, inflammatory cytokines, hyperglycemia, and microbial stimuli can induce changes that influence subsequent generations of myeloid cells [53]. Thus, an inflammatory exposure does not necessarily cease to matter when the initiating signal disappears [55].

The molecular basis of this persistence lies in the close relationship between epigenetic regulation and cellular metabolism [56]. Changes in histone modifications and chromatin accessibility can leave inflammatory loci more readily available for transcription during subsequent stimulation [57]. At the same time, activation of innate immune cells is accompanied by substantial metabolic remodeling. Glycolysis, mitochondrial oxidative metabolism, fatty acid handling, amino acid metabolism, and the mevalonate pathway are not simply altered because an inflammatory cell requires additional energy. These pathways generate metabolites that influence signaling and chromatin-modifying enzymes, thereby helping determine the cell’s subsequent behavior. This relationship between metabolism and epigenetic state is particularly relevant to atherosclerosis because many of the exposures driving the disease are themselves metabolic [58]. Hypercholesterolemia, diabetes, obesity, and insulin resistance do not merely coexist with vascular inflammation; they alter the environment in which immune cells are generated and activated. A monocyte produced during prolonged metabolic inflammation may reach the carotid plaque with a response threshold different from that of a cell generated under metabolically quiescent conditions. Once within the plaque, the recruited monocyte encounters a second layer of metabolic and inflammatory conditioning imposed by the local tissue environment [59,60].

Oxygen tension, lipid composition, oxidative stress, nutrient availability, and other metabolic features are not uniformly distributed across the fibrous cap, shoulder regions, necrotic core, neovascularized areas, and deeper vascular compartments [61]. These spatial differences are accompanied by distinct cellular and molecular programs within individual plaques [62]. Systemic conditioning and local instruction should instead be viewed as consecutive and interacting processes [63]. The bone marrow influences the composition and functional state of the myeloid cells available for recruitment. In contrast, the plaque microenvironment further shapes their phenotype and function after they enter the arterial wall [64].

Diabetes, smoking, obesity, chronic kidney disease, aging, and recurrent inflammatory or infectious exposures can each influence vascular and immune biology through partly distinct mechanisms, including endothelial dysfunction, altered hematopoiesis, innate immune reprogramming, and changes in cellular metabolism [65]. These effects are unlikely to be biologically equivalent, and some may persist after the initiating exposure has diminished or conventional risk-factor control has been achieved [66]. Such persistence may help explain why measurements of current risk-factor burden do not fully account for the inflammatory heterogeneity observed among patients with established atherosclerosis [67]. The metabolic and inflammatory state measured at a single point in time provides only a partial account of the biological environment in which an atherosclerotic plaque developed and evolved [68].

A carotid plaque obtained at endarterectomy represents a late snapshot of a biological process that has evolved over many years. Histological examination can define its current structural and cellular composition, including macrophage and lymphocyte infiltration, lipid-rich necrotic areas, intraplaque hemorrhage, calcification, neovascularization, and fibrous cap architecture [5]. These features, however, represent the cumulative result of cellular and molecular processes that preceded removal of the lesion rather than a direct record of their temporal sequence. Some of these processes arise within the arterial wall. In contrast, others are influenced by systemic inflammatory and metabolic signals and by the prior conditioning of circulating myeloid cells and their bone marrow progenitors [11]. The excised plaque may therefore retain the biological consequences of earlier inflammatory and metabolic exposures, even when the nature, timing, and relative contributions of those exposures can no longer be reconstructed from the tissue itself [66].

This view also raises a clinically relevant question. Two patients may have the same degree of carotid stenosis and broadly similar plaque morphology, yet have reached that state through different immunological trajectories. One lesion may be dominated by persistent recruitment of metabolically primed myeloid cells, another by inefficient resolution and efferocytosis, and another by a more organized adaptive immune response. These possibilities cannot be distinguished by stenosis alone and may not be adequately represented by a single circulating inflammatory biomarker.

These observations indicate that the immune state of an established carotid plaque cannot be understood as the result of a single exposure or a single inflammatory pathway. Instead, it reflects the cumulative and interacting effects of systemic risk factors, hematopoietic conditioning, vascular injury, and local tissue-specific processes that develop over time, some of which may persist beyond the initiating stimulus and influence the response to subsequent exposures [61]. The principal determinants of this immunological biography, together with their potential cellular imprint and measurable consequences, are outlined in Table 1.

### Literature Search Strategy

The literature supporting this review was identified through a structured search of PubMed, supplemented by examination of the reference lists of relevant original studies and reviews. The search was designed to capture the principal biological domains discussed in the manuscript rather than to address a single predefined intervention or outcome. Combinations of Medical Subject Headings (MeSH) and free-text terms were used, including “carotid atherosclerosis”, “carotid plaque”, “atherosclerotic plaque”, “plaque vulnerability”, “vascular inflammation”, “trained immunity”, “innate immune memory”, “monocyte”, “macrophage”, “efferocytosis”, “NLRP3”, “neutrophil extracellular traps”, “T cell”, “B cell”, “adaptive immunity”, “immune memory”, “T-cell receptor”, “TCR clonality”, “B-cell receptor”, “tertiary lymphoid structures”, “single-cell RNA sequencing”, “spatial transcriptomics”, “spatial proteomics”, and “multi-omics”. Searches addressing the translational component additionally included terms related to “carotid endarterectomy”, “symptomatic carotid stenosis”, “plaque imaging”, “circulating biomarkers”, and “stroke”. Boolean operators (AND/OR) were used to combine disease-specific terms with individual immune mechanisms, cell populations, and analytical technologies.

Priority was given to original studies of human carotid atherosclerosis, particularly those incorporating carotid endarterectomy tissue, paired blood–plaque analyses, single-cell or immune-repertoire profiling, and spatially resolved approaches. Mechanistic studies in experimental models and atherosclerosis studies at other vascular sites were included when they provided biological information not yet available from human carotid tissue. Recent reviews and consensus documents were used primarily to establish broader biological or clinical context and to identify additional primary studies. Particular attention was given to contemporary literature, while earlier landmark studies were retained for concepts central to interpreting plaque immunobiology. Publications were assessed for their relevance to the review’s conceptual framework, biological and methodological rigor, and direct contribution to understanding immune history, cellular state, clonality, spatial organization, or plaque vulnerability. Because the purpose of this article was integrative and hypothesis-generating rather than to estimate a pooled treatment effect or diagnostic performance, the literature search was not conducted as a formal systematic review or meta-analysis. The final PubMed search was performed on 2 October 2026.

## 2. Cellular Memory and Clonal Immunity: The Adaptive Immune Architecture of Carotid Atherosclerosis

**Figure 2:**
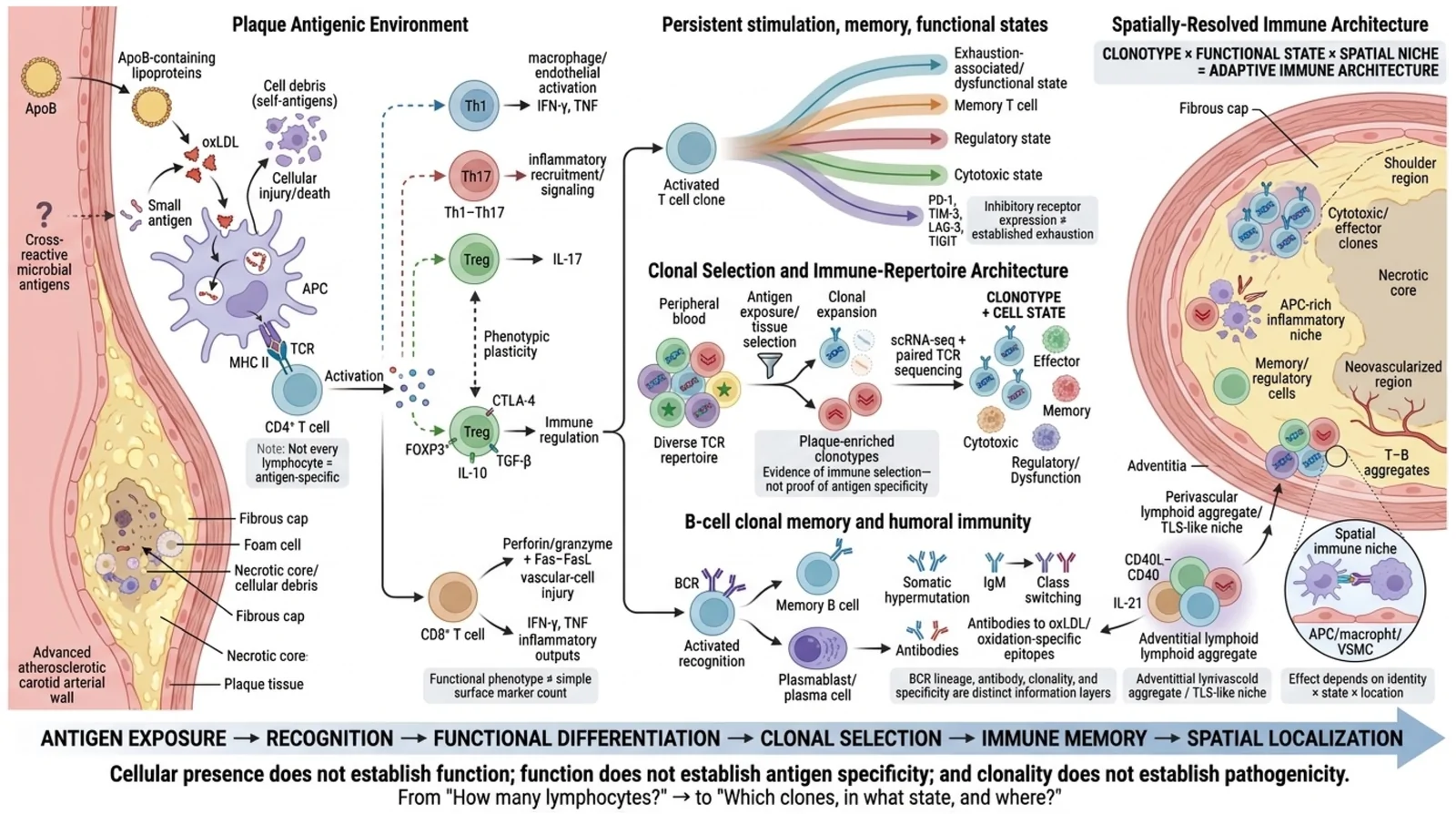
Adaptive Immune Memory and Clonal Selection in the Carotid Plaque: From Antigen Recognition to Functional and Spatial Diversification. **Abbreviations:** APC, antigen-presenting cell; ApoB, apolipoprotein B; BCR, B-cell receptor; CD4, cluster of differentiation 4; CD8, cluster of differentiation 8; CD40, cluster of differentiation 40; CD40L, CD40 ligand; CTLA-4, cytotoxic T-lymphocyte-associated protein 4; Fas, Fas cell surface death receptor; FasL, Fas ligand; FOXP3, forkhead box P3; IFN-γ, interferon gamma; IgM, immunoglobulin M; IL, interleukin; LAG-3, lymphocyte-activation gene 3; MHC II, major histocompatibility complex class II; oxLDL, oxidized low-density lipoprotein; PD-1, programmed cell death protein 1; scRNA-seq, single-cell RNA sequencing; TCR, T-cell receptor; TGF-β, transforming growth factor beta; Th1, T helper 1 cell; Th17, T helper 17 cell; TIGIT, T-cell immunoreceptor with immunoglobulin and ITIM domains; TIM-3, T-cell immunoglobulin and mucin-domain containing-3; TLS, tertiary lymphoid structure; TNF, tumor necrosis factor; Treg, regulatory T cell; VSMC, vascular smooth muscle cell. Created by Urbanowicz T, on 3 October 2026 with www.figurelabs.ai (ID: FL-PUB-20261003-XKCJDJ).

Lymphocyte accumulation within human atherosclerotic plaques has been recognized for decades, but the presence of T cells alone provides limited information about the nature of the adaptive immune response. Identification of CD4+ or CD8+ T cells in carotid endarterectomy specimens establishes their participation in the plaque immune environment. Still, it does not distinguish passive recruitment from local activation, antigen-driven responses, or the persistence of previously antigen-experienced populations [83,84]. These distinctions are biologically important. Plaque T cells encompass heterogeneous functional states, including activated, effector, memory, regulatory, cytotoxic, and exhaustion-associated phenotypes, several of which can now be resolved at the single-cell level in human carotid lesions. Evidence of cellular activation should not, however, be considered synonymous with antigen specificity, just as antigen recognition does not necessarily establish substantial local clonal expansion. These processes represent related yet distinct levels of adaptive immunity that require different experimental approaches to demonstrate. Recent integration of single-cell transcriptomics with T-cell receptor sequencing has begun to resolve this hierarchy by linking transcriptional state to individual clonotypes and identifying plaque-enriched clonal expansions with signatures consistent with recent TCR engagement [85]. The adaptive immune compartment of the carotid plaque should therefore be interpreted across successive levels—from cellular abundance and functional state to antigen recognition and, ultimately, clonal selection. Defining these levels separately will be essential for distinguishing nonspecific inflammatory recruitment from adaptive immune responses that are selectively maintained or expanded within the diseased arterial wall. Figure 2 illustrates this progression and places functional phenotype and spatial localization within the same framework.

Innate immune training can alter the magnitude and threshold of subsequent inflammatory responses without requiring antigen-specific recognition [86]. In atherosclerosis, which develops over decades, persistent and recurrent exposure to modified lipoproteins, products of cellular injury and death, inflammatory proteins, and potentially cross-reactive microbial antigens provides multiple opportunities for antigen-specific responses to emerge and evolve [87,88]. The adaptive immune compartment of an advanced carotid plaque should therefore not be viewed simply as another component of a generalized inflammatory infiltrate. Rather, its composition may reflect the cumulative effects of antigen recognition, clonal selection, differentiation, and the establishment or persistence of immunological memory [89].

CD4+ T cells occupy a central position within the adaptive immune compartment of the atherosclerotic plaque [90]. Among their functional states, Th1-associated responses remain the most extensively characterized [91]. Through the production of IFN-γ and related effector signals, Th1 cells can sustain macrophage activation, increase antigen-presenting capacity, promote endothelial inflammatory responses, and modify VSMC function [92]. These effects occur within a tissue environment already shaped by lipid accumulation, oxidative stress, cellular injury, and innate immune activation. Th1 immunity should therefore not be considered an isolated inflammatory pathway. It may reinforce and perpetuate inflammatory programs initiated through innate mechanisms, providing an important interface between antigen-specific adaptive immunity and the broader inflammatory biology of the plaque [93].

Th17-associated immunity is more difficult to position within a simple protective–pathogenic framework. IL-17 can promote chemokine production and leukocyte recruitment and has been associated with vascular inflammation and features of plaque vulnerability in human atherosclerosis [94,95]. Its role, however, appears to be context-dependent. Experimental studies have produced divergent results, suggesting that the consequences of IL-17 signaling may vary according to disease stage, cellular source, the surrounding cytokine environment, and the state of the vascular wall [96]. The phenotypic plasticity of plaque T cells further increases this complexity. Human atherosclerotic lesions contain heterogeneous CD4+ T-cell states that do not necessarily conform to the canonical lineage definitions derived from experimental models [93]. Mixed or transitional transcriptional programs, including Th1/Th17- and Treg/Th17-associated features, indicate that differentiation within chronically inflamed vascular tissue remains dynamic rather than fixed [97]. In advanced human carotid disease, characterization of functional and transcriptional states may therefore provide greater biological resolution than assignment of individual CD4+ T cells to rigid lineage categories.

Regulatory T cells (Tregs) provide an important counterbalance to effector immunity by maintaining peripheral tolerance and limiting excessive inflammatory responses. In atherosclerosis, their protective functions extend beyond direct suppression of effector T cells to include modulation of antigen-presenting cell and macrophage responses via IL-10, TGF-β, CTLA-4, and other contact-dependent and soluble regulatory mechanisms [98,99,100]. In an established plaque, however, the biological significance of the Treg compartment cannot be inferred solely from the number of FOXP3-expressing cells. The surrounding tissue environment shapes Treg phenotype and function, and the combination of persistent inflammation, hyperlipidemia, hypoxia, and metabolic stress may influence their stability, localization, and suppressive capacity [101]. Under these conditions, some Tregs may retain FOXP3 while acquiring transcriptional features associated with effector T-cell programs. In contrast, loss of lineage stability can generate ex-Treg populations with diminished regulatory activity [102]. Importantly, recent single-cell analysis of human carotid plaques suggests that this plasticity is not restricted to experimental models: plaque FOXP3+ Tregs from patients with symptomatic carotid disease displayed pro-inflammatory characteristics and transcriptional features consistent with transition toward Th17- and Th9-like states [93]. Thus, in advanced atherosclerosis, the presence of FOXP3 should not automatically be equated with preserved regulatory function; the functional state and stability of the Treg compartment may be more informative than its numerical abundance alone. The balance between regulatory and effector T-cell immunity should be considered primarily as a functional relationship rather than a simple numerical ratio. The biological contribution of the Treg compartment depends not only on its abundance, but also on the stability of the regulatory phenotype and preservation of suppressive function [103]. Conversely, the impact of effector T cells is determined by their activation and differentiation state, antigen specificity, clonal expansion, tissue localization, and interactions with other immune and vascular cells. This distinction is particularly important when interpreting human plaque studies. Quantification of CD3+, CD4+, CD8+, or FOXP3+ cells provides valuable information on the cellular composition of the lesion. Still, cellular abundance alone cannot establish the functional direction or magnitude of the local adaptive immune response [104]. Integration of conventional immunophenotyping with transcriptional profiling, functional characterization, and, where possible, T-cell receptor repertoire analysis is therefore required to distinguish cellular presence from regulatory competence or antigen-driven effector activity [97].

CD8+ T cells add a further layer of complexity to the adaptive immune response within the atherosclerotic plaque. Once activated, cytotoxic T cells can induce target-cell injury through perforin–granzyme-dependent mechanisms and death-receptor pathways, including Fas–FasL. At the same time, the release of IFN-γ and TNF can extend their influence beyond direct cytotoxicity [105]. Endothelial cells, VSMCs, macrophages, and other plaque-resident cells may therefore be affected either directly or indirectly by the inflammatory environment generated by activated CD8+ T cells [106]. Importantly, these effects are unlikely to be spatially equivalent throughout the lesion. Cytotoxic activity occurring close to the luminal endothelium or within a thin, inflamed fibrous cap could have different structural consequences from comparable activity within deeply fibrotic or calcified regions. Whether particular clonally expanded CD8+ T-cell populations preferentially localize to such vulnerable microdomains remains unresolved and is an important question for spatial studies of human carotid disease [107,108]. Persistent antigenic stimulation introduces an additional dimension to CD8+ T-cell biology. Prolonged TCR engagement and chronic inflammatory signaling can drive progressive functional adaptation, accompanied by altered transcriptional, epigenetic, and metabolic programs, and by increased expression of inhibitory receptors, including PD-1, TIM-3, LAG-3, and TIGIT [109,110]. These features are frequently discussed in the context of T-cell exhaustion, but their interpretation in atherosclerotic tissue warrants caution. Expression of an individual inhibitory receptor, particularly PD-1, does not, by itself, establish a functionally exhausted state [111]. PD-1 can accompany recent activation, tissue adaptation, or intermediate states that retain substantial proliferative and effector capacity. In contrast, more established exhaustion is characterized by the integration of functional impairment with sustained inhibitory receptor expression and characteristic transcriptional, epigenetic, and metabolic programs [112]. A PD-1+ plaque T cell should therefore not automatically be classified as exhausted. Depending on its broader phenotype and tissue context, it may represent an activated cell, an adaptive response to persistent antigenic stimulation, a progenitor or intermediate exhaustion-associated state, or a more terminally dysfunctional population. Distinguishing these states will require functional and molecular characterization beyond conventional checkpoint-receptor phenotyping.

Attenuation of T-cell effector function may, in some settings, limit immune-mediated tissue injury, but this should not be equated with restoration of immune homeostasis. Persistent functional impairment may coexist with unresolved low-grade inflammation, particularly when regulatory and pro-resolving mechanisms are themselves compromised. Moreover, inhibitory pathways are unlikely to have uniform consequences across the heterogeneous T-cell populations present within chronically inflamed tissue. Their biological significance depends on the differentiation state of the responding cell, the duration and strength of antigenic stimulation, and the surrounding metabolic and inflammatory environment [111,113]. Plaque lymphocytes should therefore not be assigned to a simple binary classification of “activated” or “exhausted.” A more informative approach will require integrating transcriptional and epigenetic states with metabolic competence, proliferative capacity, cytokine production, effector function, and TCR clonality [114,115]. Only within such a multidimensional framework can inhibitory-receptor expression be interpreted as evidence of recent activation, adaptive restraint, progenitor or intermediate exhaustion-associated states, or more advanced functional dysfunction.

ApoB-containing lipoproteins provide one of the clearest mechanistic links between the metabolic substrate of atherosclerosis and antigen-specific adaptive immunity. ApoB-derived peptides can be processed and presented by MHC class II molecules and recognized by CD4+ T cells. More recent immunopeptidomic studies have further shown that ApoB100-derived peptides are presented by HLA-DR molecules within human atherosclerotic plaques, providing direct evidence that components of retained lipoproteins can enter local antigen-presentation pathways [89]. Lipoprotein modification may further broaden the antigenic landscape of the lesion. Oxidation and other chemical modifications generate oxidation-specific epitopes and may alter how lipoprotein-derived material is recognized by the immune system [116]. The biological significance of ApoB-specific immunity, however, cannot be inferred from antigen recognition alone. ApoB-reactive CD4+ T cells display considerable functional heterogeneity and may exhibit regulatory, memory-like, or inflammatory transcriptional programs depending on disease context [117]. Experimental studies suggest that ApoB-reactive populations with an initially regulatory phenotype can acquire mixed Th1/Th17-like characteristics during the progression of atherosclerosis [118]. Consistent with this concept, human studies have identified a shift from regulatory to memory-like or inflammatory programs among ApoB-reactive CD4+ T cells in individuals with more advanced cardiovascular disease [87]. These observations raise the possibility that recognition of the same self-antigen may have different immunological consequences at different stages of atherosclerosis. Whether this reflects true lineage conversion of individual ApoB-reactive clones in humans, differential expansion of pre-existing subsets, or both remains unresolved. This distinction is particularly important in carotid disease, where direct characterization of antigen-specific T-cell states within anatomically defined plaque regions remains limited. Antigen specificity and functional phenotype should therefore be considered as related but distinct dimensions of adaptive immunity: identifying what a T cell recognizes does not, by itself, establish whether its contribution to the plaque is regulatory, neutral, or pro-inflammatory.

The recognition of modified self-antigens raises a broader question as to where plaque immunity lies along the continuum between physiological immune surveillance, loss of tolerance, and autoimmunity. Atherosclerosis is not conventionally classified as an autoimmune disease, and application of this terminology to the disease as a whole would overlook its fundamental metabolic, vascular, and innate immune components. Nevertheless, several features of adaptive immunity within atherosclerotic tissue overlap with processes observed in autoimmune disease, including the persistent availability of self-derived antigens, MHC-restricted T-cell recognition, repeated antigenic stimulation, autoantibody formation, and local clonal expansion of antigen-experienced lymphocytes [119]. Human carotid plaque studies have strengthened this concept by demonstrating plaque-enriched T-cell clonotypes with evidence of recent TCR engagement. At the same time, immunopeptidomic analyses have directly identified ApoB-derived peptides presented by HLA-DR in atherosclerotic tissue [120]. These observations, however, should not be interpreted as evidence that autoimmunity uniformly initiates atherosclerosis. A more plausible framework is that loss or modification of self-tolerance may emerge at different stages of an already evolving vascular disease and subsequently become one of several mechanisms that sustain or reshape plaque inflammation [118,121]. The biological significance of an autoreactive response is also likely to depend on the antigen involved, the phenotype of the responding lymphocyte, the local antigen-presenting environment, and the stage of lesion development. Indeed, recognition of self-derived epitopes may support homeostatic or regulatory immunity in one context and inflammatory amplification in another [122]. The presence of autoreactive T cells or autoantibodies should therefore be regarded as evidence of immune recognition rather than, by itself, proof of pathogenic autoimmunity. Establishing causality will require demonstration that a defined antigen-specific response precedes, modifies, or perpetuates vascular injury rather than simply arising as a consequence of chronic tissue damage.

Each T-cell clone carries a rearranged T-cell receptor (TCR) sequence that serves as a molecular identifier for its clonal lineage. This makes TCR repertoire analysis fundamentally different from conventional assessment of lymphocyte abundance: it asks not only how many T cells are present, but whether particular clones have been selectively enriched within the lesion. A broadly diverse repertoire and one dominated by a limited number of expanded clonotypes represent biologically different immune architectures. In human atherosclerotic plaques, expanded T-cell clonotypes have been identified, and paired blood–plaque analyses indicate that some expansions are preferentially enriched within the diseased arterial wall [90]. Such enrichment is consistent with selective recruitment, retention, local proliferation, or repeated antigen-driven stimulation, but clonality alone cannot distinguish among these possibilities and, critically, does not identify the antigen responsible [123]. Clonal expansion should therefore be regarded as evidence of biological selection rather than proof of antigen specificity. The interpretative value of TCR sequencing increases substantially when the clonotype is linked to the cellular phenotype. Integration of single-cell transcriptomics with paired TCR sequencing enables the determination of whether members of the same clone occupy effector, cytotoxic, regulatory, memory, or dysfunction-associated states, and whether related clonotypes span more than one transcriptional phenotype. Comparison with matched peripheral blood adds a second level of resolution. A clonotype that is uncommon in the circulation but markedly enriched within the plaque provides stronger evidence of tissue-associated selection than a highly abundant circulating clone that is proportionately represented within the lesion. The next step is spatial. If clonotype, transcriptional state, and anatomical localization can be resolved simultaneously, expanded T-cell populations could be assigned to specific plaque microenvironments, including the fibrous cap, shoulder regions, necrotic core interface, adventitia, or perivascular lymphoid structures [124]. At that point, the central question changes from how many lymphocytes are present to which clones are present, what functional states they occupy, and where within the plaque those states are expressed.

B cells have traditionally received less attention than T cells and macrophages in atherosclerosis, although their contribution to adaptive immunity extends well beyond antibody production. They can function as antigen-presenting cells, provide regulatory or inflammatory cytokine signals, influence T-cell responses, and differentiate into memory B cells, plasmablasts, and long-lived antibody-secreting populations [125]. As with T cells, therefore, the designation “B cell” describes a lineage rather than a uniform functional state. Different B-cell populations may have opposing effects in atherosclerosis: some participate in immune regulation and the clearance of oxidation-associated structures, whereas others may sustain antigen presentation, T-cell activation, and humoral responses to vascular antigens [126]. The presence of antibodies that recognize oxidized LDL and other oxidation-specific epitopes provides clear evidence that humoral recognition accompanies atherosclerosis. Still, the biological significance of these antibodies is not uniform [127]—isotype, affinity, epitope specificity, and the cellular origin of the response all influence their function. Naturally occurring IgM antibodies that recognize oxidation-specific epitopes can facilitate the clearance of oxidized lipids and apoptotic material and are generally associated with atheroprotective mechanisms.

In contrast, class-switched responses may have different consequences, including the potential formation of immune complexes and the engagement of Fc receptors or complement pathways [128,129]. Consequently, measurement of a circulating antibody concentration captures only one endpoint of a considerably more complex immune process. A more informative question is how the underlying B-cell response is organized. A serum antibody measurement cannot establish where the responsible clone was activated, whether it underwent germinal-center selection and affinity maturation, whether related cells persist within the memory compartment, or whether antibody-secreting descendants are present within the arterial wall. B-cell receptor (BCR) repertoire analysis provides a means of reconstructing part of this history. Immunoglobulin V(D)J sequences can identify clonally related cells, while patterns of somatic hypermutation and class switching provide evidence of antigen-experienced maturation [120,130]. When BCR sequencing is integrated with single-cell transcriptomics, clonotype can be linked directly to cellular state, allowing related cells to be assigned to naïve, activated, memory, germinal-center-like, plasmablast, or plasma-cell programs [93]. Paired analysis of blood and plaque could then determine whether particular B-cell lineages are merely represented in the systemic repertoire or are selectively enriched within diseased vascular tissue. Ultimately, extending this approach to spatially resolved analyses could establish whether clonally related B cells and antibody-secreting cells occupy defined immune niches within or adjacent to the plaque.

These observations reveal several distinct levels of adaptive immunity within the plaque, ranging from cellular infiltration and differentiation to antigen recognition, clonal selection, and persistence of antigen-experienced populations. Keeping these levels separate is essential because the presence of a lymphocyte does not establish its function, and clonal expansion alone does not identify the antigen driving that expansion. Table 2 places the major adaptive immune populations and processes within this hierarchy and highlights the limits of what can be inferred from each.

The arterial adventitia provides a particularly intriguing anatomical setting in which local adaptive immune selection may occur. Chronic vascular inflammation can be accompanied by progressive organization of adventitial lymphocyte aggregates, ranging from relatively simple cellular clusters to tertiary lymphoid structures (TLS) with features resembling those of secondary lymphoid organs [143]. In their more developed forms, these structures may contain spatially segregated T- and B-cell compartments, antigen-presenting cells, follicular dendritic-cell networks, specialized vasculature with high endothelial venule-like properties, and B-cell follicles capable of supporting germinal-center-associated responses [144]. Their development challenges a strictly lymph-node-centered view of adaptive immunity in atherosclerosis. Rather than functioning solely as a passive outer layer of the vessel wall, the adventitia may provide a local immune niche in which antigen presentation, lymphocyte recruitment, T–B-cell cooperation, and clonal selection occur in close anatomical proximity to the diseased artery [145]. Recent human data make this possibility increasingly relevant. Spatially resolved single-cell analysis of human atherosclerotic tissue has identified plaque-associated tertiary lymphoid organ-like structures containing abundant B cells with expanded and diversified B-cell receptor repertoires, providing evidence that organized vascular immune niches may support local or regional B-cell selection [142]. Earlier histopathological studies of human coronary arteries similarly identified distinct stages of adventitial lymphoid organization and demonstrated an association between more advanced structures and more advanced or unstable atherosclerotic lesions [146]. These observations raise the possibility that a lymphocyte participating in an organized perivascular immune niche may have a fundamentally different biological relationship to the plaque from that of a circulating lymphocyte transiently recruited through an inflamed endothelium. Much of the mechanistic understanding of artery tertiary lymphoid organs—including compartmentalized T- and B-cell responses, germinal-center activity, high endothelial venules, and local antigen-driven immune selection—derives from hyperlipidemic mouse models and should not be transferred uncritically to human carotid atherosclerosis. Even in human tissue, the presence of an organized lymphoid aggregate does not, by itself, establish sustained antigen-specific activity or demonstrate that the structure causally contributes to plaque progression. The prevalence, maturation state, antigenic repertoire, and functional consequences of TLS in human carotid disease remain incompletely defined [147]. Nevertheless, their existence provides a compelling anatomical framework in which persistent vascular inflammation could evolve from diffuse leukocyte accumulation toward locally organized adaptive immunity. Defining whether such niches support antigen presentation, clonal selection, B-cell maturation, and persistence of disease-relevant lymphocyte populations should therefore be an important objective of spatial and repertoire-based studies of human carotid atherosclerosis.

Peripheral blood provides the most accessible window into adaptive immunity, but it should not be assumed to reproduce the immune architecture of an individual atherosclerotic plaque. Circulating and tissue-resident lymphocyte compartments are related but not interchangeable. Paired analyses of human carotid plaques and peripheral blood have demonstrated plaque-enriched T-cell populations and clonotypes that are only weakly represented in the circulation, indicating that systemic measurements may substantially underestimate immune populations selectively accumulated within the diseased arterial wall [93]. A similar principle appears to apply to humoral immunity, as B-cell receptor repertoires recovered from human atherosclerotic plaques can differ markedly from those detected in peripheral blood, with reduced repertoire diversity and an enrichment of selected B-cell clones within vascular tissue [148]. Conversely, the numerical prominence of a lymphocyte population in the blood does not establish its participation in the local plaque response. Entry into the plaque exposes lymphocytes to a tissue environment characterized by persistent antigenic stimulation, inflammatory cytokines, altered nutrient and oxygen availability, lipid accumulation, oxidative stress, and sustained interactions with myeloid and vascular cells. These signals can impose transcriptional and functional states that are not evident from the analysis of phenotypically related circulating cells [90,149]. Thus, even cells belonging to the same lineage—and potentially the same clonotype—cannot necessarily be assumed to retain equivalent biological states in blood and vascular tissue. The relevant question is therefore not whether peripheral blood can serve as a substitute for the plaque, but which components of plaque immunity are reflected in the circulation and which remain compartmentalized within the arterial wall. Paired blood–plaque profiling, particularly when cellular phenotype is integrated with TCR or BCR repertoire analysis, provides a more informative strategy for distinguishing systemic immune signatures from tissue-enriched adaptive responses.

Carotid endarterectomy provides an unusual translational opportunity to interrogate advanced human atherosclerosis directly rather than infer plaque biology from circulating biomarkers alone. Importantly, the procedure can provide access not only to the lesion itself but also to matched peripheral blood and, in selected settings, to blood sampled in proximity to the diseased artery, adjacent vascular tissue, and pericarotid lymphoid compartments [150,151]. This creates the possibility of studying vascular immunity as a compartmentalized biological system. Paired plaque–blood analyses can distinguish broadly circulating immune states from cell populations, clonotypes, and molecular programs selectively enriched within the arterial wall, whereas sampling of adjacent vascular or lymphoid tissue may help determine whether these responses extend beyond the plaque into regional immune niches [152,153]. Such an approach is particularly relevant for adaptive immunity, where integration of single-cell phenotyping with TCR/BCR repertoire analysis could determine whether expanded lymphocyte clones are systemic, plaque-enriched, or shared across anatomically connected vascular and lymphoid compartments. Endarterectomy should therefore be viewed not simply as an opportunity to obtain diseased tissue, but as a clinically grounded model in which the spatial and clonal organization of human vascular immunity can be reconstructed across blood, plaque, and the surrounding immune environment.

## 3. The Spatial Immune Ecosystem of the Vulnerable Carotid Plaque

Atherosclerotic plaques are conventionally described by their cellular and structural composition: macrophages, lymphocytes, vascular smooth muscle cells, extracellular matrix, lipid deposits, calcification, and necrotic material. Although this description remains fundamental to histopathological assessment, it captures only part of the biological organization of an advanced lesion. These components are not distributed randomly. These components occupy distinct anatomical and functional niches, such that the biological significance of an immune response is determined not only by the identity and activation state of the cells involved, but also by their location within the plaque and their interactions with the surrounding cellular and molecular environment. This spatial context is particularly important when interpreting immune-cell phenotypes. A macrophage situated within the fibrous cap encounters a different extracellular matrix, lipid environment, oxygen tension, and cellular neighborhood from a macrophage positioned at the interface with the necrotic core or within an area of intraplaque hemorrhage [154,155]. Similarly, a T cell located in a plaque shoulder, in close proximity to antigen-presenting cells, may engage in a different biological process than a phenotypically similar cell dispersed within a collagen-rich, relatively quiescent region. The anatomical position of inflammation also determines its potential consequences for the vessel: immune activity immediately beneath the luminal surface or within a thin fibrous cap may directly influence structures relevant to plaque integrity, whereas inflammation within the adventitia may contribute to perivascular immune organization and communication with regional lymphoid compartments [124].

**Figure 3:**
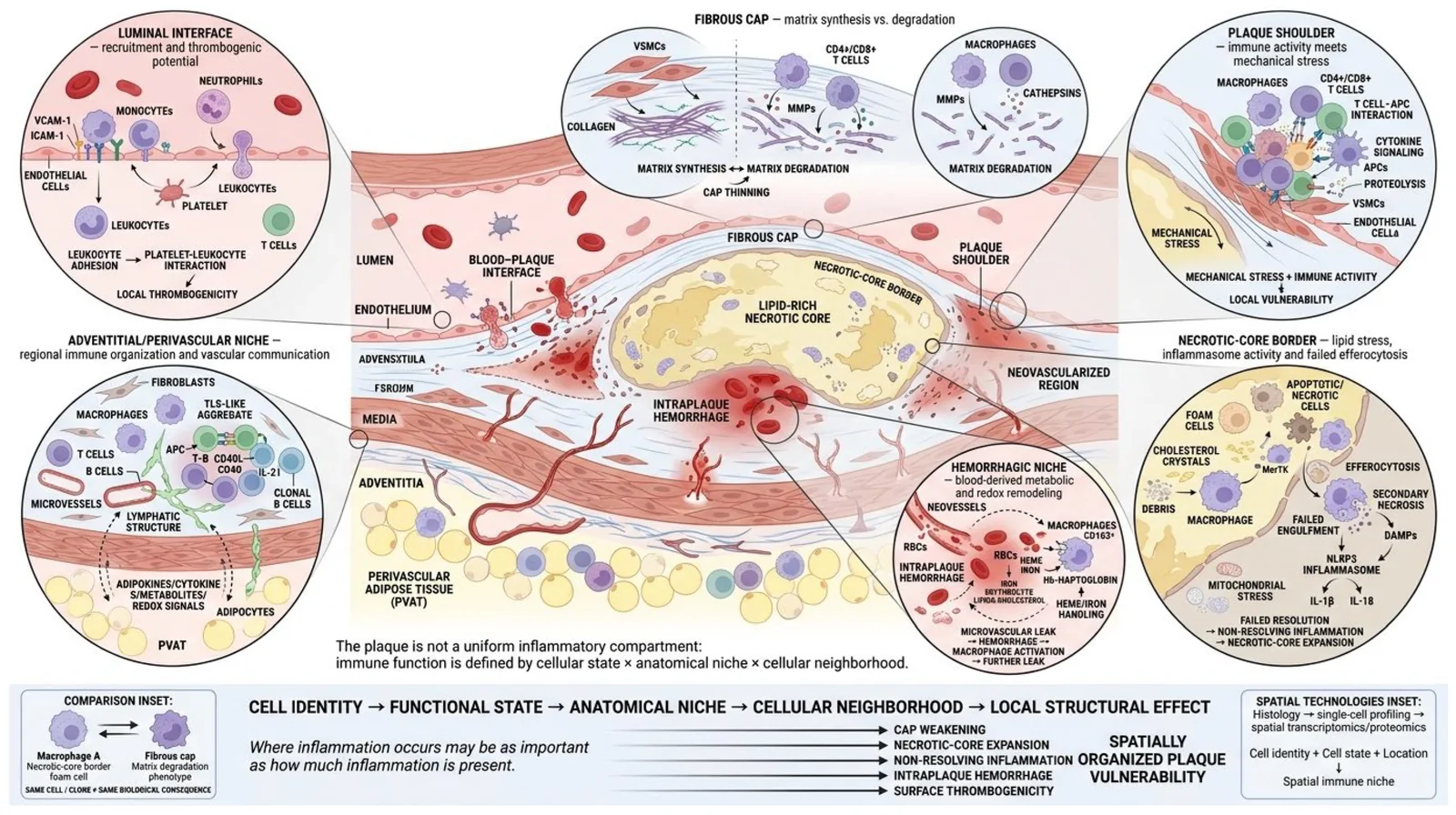
Spatial Immune Niches of the Vulnerable Carotid Plaque: Cellular Neighborhoods Linking Inflammation to Structural Instability. **Abbreviations:** APC, antigen-presenting cell; CD4, cluster of differentiation 4; CD8, cluster of differentiation 8; CD40, cluster of differentiation 40; CD40L, CD40 ligand; CD163, cluster of differentiation 163; DAMPs, damage-associated molecular patterns; Hb, hemoglobin; ICAM-1, intercellular adhesion molecule 1; IL, interleukin; MerTK, MER proto-oncogene tyrosine kinase; MMPs, matrix metalloproteinases; NLRP3, NOD-, LRR- and pyrin domain-containing protein 3; PVAT, perivascular adipose tissue; RBCs, red blood cells; TLS, tertiary lymphoid structure; VCAM-1, vascular cell adhesion molecule 1; VSMCs, vascular smooth muscle cells. Created by Urbanowicz T, on 3 October 2026 with www.figurelabs.ai (ID: FL-PUB-20261003-OKAL1Q).

The advanced carotid plaque should therefore be considered not simply as an inflammatory lesion, but as a spatially organized immune ecosystem. The fibrous cap, plaque shoulder, necrotic core interface, hemorrhagic regions, neovascularized areas, and adventitia represent distinct microenvironments in which apparently similar immune populations may acquire different functional states and exert distinct biological effects [142]. Recent spatial transcriptomic, proteomic, and metabolomic studies of human atherosclerosis are beginning to resolve this organization directly, demonstrating that molecular and cellular programs vary substantially across anatomically defined regions of the same lesion [139]. Figure 3 illustrates this spatial organization and highlights how the functional significance of an immune population may vary with its anatomical niche and interactions with neighboring immune, vascular, and stromal cells.

Carotid plaque vulnerability is ultimately expressed as a local failure of tissue integrity. Neither the total number of macrophages within a lesion nor the concentration of a circulating inflammatory biomarker determines, in isolation, whether a plaque will become clinically unstable. Cerebral embolization requires disruption of the luminal interface—through plaque rupture, erosion, or other forms of surface injury—with exposure of thrombogenic material, superimposed thrombus formation, and subsequent embolization [156,157]. The biological processes that precede such an event must therefore be interpreted in their anatomical context. This principle is particularly evident at the fibrous cap, where mechanical integrity depends on the balance between extracellular matrix synthesis and degradation and on the capacity of vascular smooth muscle cells (VSMCs) to maintain and repair the collagen-rich matrix that separates circulating blood from the thrombogenic plaque interior [158]. Inflammation within or immediately beneath this structure can shift that balance: activated macrophages produce matrix-degrading proteases, including matrix metalloproteinases and cathepsins, while inflammatory signaling can impair VSMC survival, phenotype, and matrix synthesis [159]. Importantly, the consequences of these processes depend on where they occur. Proteolytic activity within a deeply situated necrotic region may contribute to lesion remodeling, whereas comparable activity concentrated within a thin, inflamed fibrous cap has more immediate implications for structural failure. Human carotid studies support this spatial interpretation: ruptured plaques show increased accumulation and activation of macrophages and T cells within the cap, along with loss of VSMCs, while spatial transcriptomic analyses demonstrate that rupture-prone regions possess molecular programs distinct from those of more stable regions within the same lesion [124]. The advanced plaque should therefore not be viewed as a uniform inflammatory compartment. The luminal interface, fibrous cap, plaque shoulder, necrotic core border, neovascularized and hemorrhagic regions, media, and adventitia constitute biologically distinct niches in which immune, vascular, and stromal cells encounter different structural, metabolic, and inflammatory conditions [160,161]. Plaque vulnerability consequently reflects not simply which immune cells are present, but where they are located, the states they occupy, the cells with which they interact, and the local tissue structures on which their effector functions act [162]. Table 3 summarizes these spatial immune niches in human carotid plaque and relates their characteristic cellular interactions and biological processes to mechanisms that may promote or restrain local plaque instability.

Clonal expansion identifies immune selection, but it does not establish where within the plaque that selection becomes biologically consequential. A T-cell clonotype distributed sparsely throughout an artery is unlikely to have the same significance as the same clonotype concentrated within a macrophage-rich shoulder, adjacent to antigen-presenting cells, or immediately beneath a structurally compromised fibrous cap. TCR sequencing can identify expansion and tissue enrichment, but spatial localization adds a separate dimension by placing the selected clone within the cellular and structural context in which its effector functions are expressed [142]. The convergence of single-cell transcriptomics, paired TCR sequencing, and spatial transcriptomic or proteomic technologies offers an opportunity to move beyond inventories of plaque lymphocytes toward reconstruction of clonotype–state–niche relationships. The relevant question is no longer simply which clones are present, but whether selected clonotypes reproducibly occupy microenvironments associated with antigen presentation, vascular injury, matrix remodeling, or tissue repair. Establishing such relationships in human carotid disease would provide a substantially stronger basis for interpreting clonal expansion than repertoire analysis alone [124].

The same spatial principle applies to the myeloid compartment. The traditional representation of plaque macrophages as a relatively uniform inflammatory population has been superseded by single-cell studies that reveal a continuum of transcriptional and functional states [175,176]. Recruited monocyte-derived populations coexist with macrophages displaying lipid-handling, inflammatory, interferon-responsive, proliferative, resident-like, and tissue-remodeling programs, none of which can be adequately accommodated within the conventional M1/M2 classification [177]. Importantly, these states are spatially organized rather than randomly distributed. Recent integration of single-cell and spatial transcriptomic data from human carotid plaques has identified distinct macrophage territories and transitional programs, including spatial relationships between inflammatory HMOX1-expressing macrophages and TREM2-associated lipid-handling populations at the fibrous cap–necrotic core interface [68]. Thus, macrophage identity in an advanced plaque is determined not only by lineage or developmental origin, but also by the sequence of environments through which the cell has passed.

This distinction is particularly relevant to recruited monocytes. CCR2-dependent trafficking remains an important route by which inflammatory monocytes enter developing atherosclerotic lesions, especially at vascular sites characterized by endothelial activation and chemokine production [178,179]. These cells do not necessarily arrive immunologically naïve. Their functional state may already have been shaped by hyperlipidemia, systemic inflammation, metabolic disease, or persistent reprogramming of myeloid progenitors prior to recruitment to the arterial wall [28]. Entry into the plaque then exposes recruited monocytes to a second layer of instruction imposed by the local tissue environment. Resident arterial macrophages, in contrast, can be maintained through local persistence and self-renewal and are adapted to tissue surveillance and homeostatic functions, although these distinctions become progressively less clear during atherogenesis as recruited monocyte-derived macrophages acquire tissue-adapted transcriptional programs [180,181,182]. In advanced human atherosclerosis, however, the boundary between recruited and resident-like macrophages is unlikely to be absolute, and transcriptional phenotype should not be interpreted as a direct surrogate for cellular ontogeny. The broader point is more important: macrophages that appear similar by conventional histology may differ substantially in developmental history, transcriptional state, metabolic adaptation, and functional potential.

Few regions illustrate this principle more clearly than the interface with the necrotic core. Macrophages occupying this territory are simultaneously exposed to modified lipoproteins, cholesterol crystals, apoptotic and necrotic cells, hypoxia, oxidative stress, and profound changes in nutrient availability [183]. Cells initially recruited to sequester lipids and clear cellular debris may progressively lose their capacity to maintain local homeostasis as these stresses accumulate. Efferocytosis is central to this transition. Efficient recognition and removal of apoptotic cells ordinarily prevent the loss of membrane integrity and limit the release of intracellular danger signals, while promoting pro-resolving macrophage programs [184]. In advanced atherosclerosis, however, efferocytosis becomes increasingly ineffective. Impaired recognition and uptake of apoptotic cells, including disruption of MerTK-dependent signaling, permit secondary necrosis and contribute to the expansion of the necrotic core and the persistence of non-resolving inflammation [185,186]. The necrotic-core border should therefore not be viewed merely as a site of macrophage accumulation. It represents a functional transition zone in which lipid handling, cell clearance, inflammatory signaling, and tissue repair compete—and in advanced disease increasingly fail to remain balanced.

Mer tyrosine kinase (MerTK) occupies a central position in macrophage efferocytosis, coupling recognition of apoptotic cells to their engulfment while simultaneously favoring the resolution of inflammation. In advanced atherosclerosis, this protective pathway can be disrupted by proteolytic cleavage of the receptor, generating soluble MerTK and reducing the capacity of lesional macrophages to clear dying cells [187,188]. Importantly, this mechanism has been demonstrated directly in human carotid plaques, where MerTK shedding is particularly evident in tissue surrounding the necrotic core and is associated with plaque necrosis and ischemic presentation [166]. The consequence extends well beyond the passive accumulation of cellular debris. Apoptotic cells that escape timely clearance eventually lose membrane integrity and undergo secondary necrosis, releasing intracellular proteins, lipids, nucleic acids, and other damage-associated signals into a microenvironment in which mechanisms of inflammatory resolution are already compromised [189]. Necrotic-core expansion should therefore be understood not simply as the consequence of excessive cell death, but also as a failure of tissue clearance and resolution. At the same interface, macrophages encounter a convergence of signals that can engage inflammasome pathways. Cholesterol accumulation and crystallization, lysosomal injury, mitochondrial and oxidative stress, and extracellular danger signals can collectively favor NLRP3 activation, caspase-1-dependent processing of IL-1β and IL-18, and amplification of local inflammatory signaling [190,191,192,193]. These pathways should not, however, be interpreted independently of their spatial and functional context. The biological consequence of inflammasome activation depends on the cellular state in which it occurs, the surrounding vascular and immune populations, the persistence of the initiating stimulus, and the tissue’s capacity to terminate the response. Sustained inflammasome activity within a macrophage-rich environment bordering an expanding necrotic core, particularly when coupled to defective efferocytosis, therefore represents a qualitatively different biological state from transient inflammasome activation in otherwise viable tissue. In this sense, the necrotic-core interface may be viewed as a failure-of-resolution niche, in which defective clearance and persistent innate immune activation become mutually reinforcing processes.

Vascular smooth muscle cells (VSMCs) add another layer of complexity to the cellular architecture of the atherosclerotic plaque, as their contributions extend far beyond the formation of the fibrous cap. Genetic lineage-tracing studies have fundamentally revised the traditional view of VSMCs as a relatively stable, contractile population by demonstrating extensive phenotypic modulation during atherogenesis [194,195]. As disease progresses, lineage-traced VSMCs can downregulate canonical contractile markers and acquire transcriptional programs with synthetic, fibroblast-like, osteochondrogenic, lipid-handling, or macrophage-like characteristics [196,197]. This plasticity poses an important interpretative problem in human tissue: cellular identity cannot be reliably inferred from a limited set of conventional immunohistochemical markers. Cells expressing macrophage-associated proteins may, in some circumstances, originate from the VSMC lineage, whereas extensively modulated VSMC-derived cells may retain little phenotypic resemblance to their medial precursors [198]. Phenotype should therefore not be considered synonymous with ontogeny. Equally important, VSMC modulation is not intrinsically detrimental. VSMC-derived populations that populate the fibrous cap and maintain a collagen-rich extracellular matrix contribute directly to its mechanical integrity, whereas loss of these cells, impaired matrix production, apoptosis, or transition toward selected inflammatory or dysfunctional states can shift plaque architecture toward instability [199]. Experimental lineage-tracing studies further demonstrate that apparently related VSMC transitions can have divergent effects on lesion composition: regulatory programs involving KLF4, OCT4, extracellular-matrix signaling, and other determinants influence whether VSMC-derived cells accumulate within the cap, adopt matrix-producing phenotypes, or contribute to less favorable plaque compartments [200]. The biologically relevant question is therefore not whether VSMCs are globally “protective” or “pathogenic,” but which VSMC-derived state is present, what function that state retains or acquires, and where within the plaque it is positioned. As with immune cells, cellular phenotype and spatial context are inseparable: a matrix-producing VSMC-derived cell incorporated into the fibrous cap may contribute to stabilization, whereas a phenotypically modulated descendant occupying the lipid-rich or inflammatory core may participate in an entirely different biological process [201,202]. Understanding VSMCs in advanced human carotid disease will consequently require integrating cellular state with lineage-informed biology and spatial localization, rather than classification based on conventional marker expression alone.

**Figure 4:**
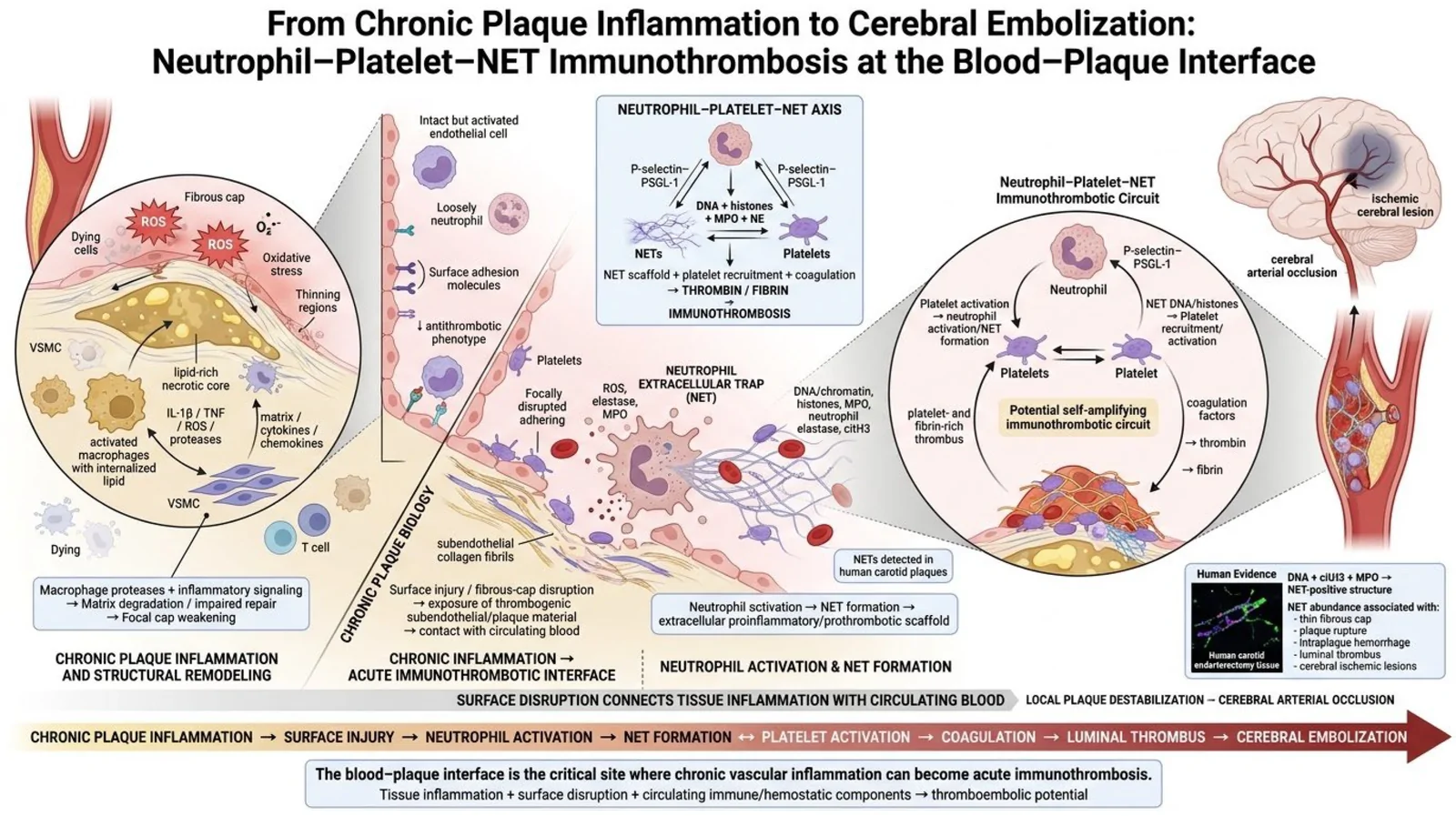
From Chronic Plaque Inflammation to Cerebral Embolization: Neutrophil–Platelet–NET Immunothrombosis at the Blood–Plaque Interface. **Abbreviations:** citH3, citrullinated histone H3; DNA, deoxyribonucleic acid; IL, interleukin; MPO, myeloperoxidase; NE, neutrophil elastase; NET, neutrophil extracellular trap; NETs, neutrophil extracellular traps; PSGL-1, P-selectin glycoprotein ligand 1; ROS, reactive oxygen species; TNF, tumor necrosis factor; VSMC, vascular smooth muscle cell. Created by Urbanowicz T, on 3 October 2026 with www.figurelabs.ai (ID: FL-PUB-20261003-LY2OKA).

Cellular interactions within the advanced plaque ultimately converge at the luminal interface, where chronic inflammation can be translated into an acute thromboembolic event. Macrophages and vascular smooth muscle cells (VSMCs) participate in reciprocal signaling rather than functioning as independent cellular compartments: macrophage-derived IL-1β, TNF, reactive oxygen species, and proteolytic enzymes can alter VSMC survival, phenotypic state, and extracellular-matrix production, whereas phenotypically modulated VSMCs generate matrix components, cytokines, and chemokines that can, in turn, influence macrophage recruitment, retention, and functional state [196]. Human carotid single-cell data support this bidirectional relationship, including IL-1β–STAT3-dependent signaling between macrophages and macrophage-like VSMCs within advanced lesions [203]. Both populations are additionally conditioned by modified lipids, dying cells, oxidative stress, and other components of the local plaque environment, making their interaction highly dependent on anatomical context. At the luminal surface, however, the biological problem changes. Endothelial activation not only regulates leukocyte recruitment through adhesion molecules and chemokine signaling but also progressively alters the antithrombotic properties of the blood–vessel interface; when endothelial integrity is compromised or thrombogenic subendothelial material is exposed, inflammatory signaling becomes directly coupled to platelet activation and coagulation [204,205,206]. This creates a narrow but biologically intense immunothrombotic interface, in which endothelial cells, platelets, monocytes, lymphocytes, and neutrophils participate in reciprocal activation. Neutrophils are particularly relevant because their biological influence may be disproportionate to their numerical representation within the plaque. Through the release of reactive oxygen species and granular proteases, neutrophils can modify endothelial and extracellular matrix integrity, while activated neutrophils can undergo NETosis and release extracellular structures composed of chromatin, histones, and granular proteins that provide a physical and biochemical platform linking innate immunity to thrombosis [171,172]. Importantly, NETs have been directly demonstrated in human carotid endarterectomy specimens, where their abundance is associated with plaque rupture, intraplaque hemorrhage, thin fibrous caps, intraluminal thrombus, and cerebral ischemic lesions [207]. Platelets should likewise be regarded as active inflammatory participants rather than merely the terminal effectors of hemostasis. Activated platelets interact directly with neutrophils and other leukocytes through adhesion systems, including P-selectin–PSGL-1 and additional receptor pairs, while releasing mediators that further promote leukocyte activation [208]. These interactions are bidirectional: platelet activation can facilitate NET formation, whereas NET-associated DNA, histones, and granular proteins provide prothrombotic surfaces that support platelet recruitment and coagulation [209,210]. The result is a potentially self-amplifying immunothrombotic circuit positioned precisely where chronic plaque biology encounters circulating blood. In carotid atherosclerosis, this transition has particular clinical importance because the endpoint is not limited to local arterial thrombosis: disruption of the plaque surface and the formation of platelet- and fibrin-rich thrombotic material create a source of emboli that can enter the cerebral circulation. Thus, the evolution from chronic plaque inflammation to an acute cerebrovascular event can be understood as a spatially restricted transition in which tissue injury, endothelial dysfunction, neutrophil activation, NET formation, platelet recruitment, and coagulation become functionally coupled. Figure 4 integrates these processes into a common immunothrombotic pathway linking local plaque destabilization to luminal thrombosis and, ultimately, cerebral embolization.

Neovascularization introduces a second vascular interface into the advanced atherosclerotic plaque and, with it, an additional route through which circulating cells and blood-derived substrates can reshape the local tissue environment. Intraplaque microvessels arise largely from the adventitial vasa vasorum, penetrate the media and expand into the intima, and frequently remain structurally immature and excessively permeable; their leakage and disruption are therefore closely linked to intraplaque hemorrhage (IPH), a hallmark of high-risk carotid plaque biology [211,212,213]. Importantly, IPH should not be regarded simply as evidence that a fragile microvessel has failed. Once erythrocytes enter the plaque, they alter its biochemical composition: erythrocyte membranes provide a substantial source of free cholesterol, whereas hemoglobin, heme, and iron introduce potent oxidative and metabolic signals capable of modifying macrophage and vascular-cell function [214,215,216]. Macrophages encountering this environment acquire phenotypes that differ from those generated by conventional lipid loading; uptake of hemoglobin–haptoglobin complexes through CD163, together with heme metabolism and iron handling, can induce distinct transcriptional and metabolic programs and influence angiogenesis, vascular permeability, inflammation, and interactions with VSMCs [186,217]. Recurrent hemorrhage may therefore generate a self-reinforcing microenvironment in which erythrocyte deposition, macrophage reprogramming, neovascular permeability, and further hemorrhage become biologically coupled [169]. In this sense, a hemorrhagic plaque has not merely accumulated blood products; it has acquired a distinct inflammatory–metabolic niche. The outer arterial wall adds a further level of spatial organization. Remodeling or disruption of the media increasingly connects the intimal lesion with an adventitia containing fibroblasts, macrophages, lymphocytes, microvessels, lymphatic structures, and neural elements, creating an immunological environment fundamentally different from that of the necrotic core or fibrous cap [144]. In some advanced arteries, persistent inflammation is accompanied by the development of organized lymphoid aggregates and tertiary lymphoid organ-like structures, providing anatomically defined sites in which antigen presentation, T–B-cell cooperation, and clonal selection may occur close to the diseased vessel [218,219]. Recent spatially resolved analysis of human atherosclerotic tissue has strengthened this concept by identifying plaque-associated tertiary lymphoid organ-like structures containing abundant B cells with expanded and diversified B-cell receptor repertoires, although whether these structures actively drive human carotid plaque progression or arise in response to chronic vascular injury remains unresolved [142]. Their existence nevertheless has important implications for the interpretation of adaptive immunity: a clonally expanded lymphocyte positioned within an organized perivascular immune structure encounters a fundamentally different network of antigen-presenting cells, B cells, stromal signals, and lymphatic or vascular trafficking pathways from an apparently similar clone residing within the intima. The adventitia should therefore not be considered merely the anatomical boundary of the plaque, but a potentially active immune compartment capable of communicating with deeper layers of the arterial wall. Perivascular adipose tissue (PVAT) extends this concept beyond the vessel itself. Adipocytes, macrophages, lymphocytes, stromal cells, microvessels, and neural elements within PVAT generate adipokines, cytokines, metabolites, and redox signals with potential access to the adjacent adventitia, while human carotid studies have associated altered pericarotid adipose-tissue characteristics with systemic inflammatory indices and vulnerable plaque features [220,221]. These observations support a bidirectional rather than exclusively luminal model of vascular inflammation, although current human data cannot establish whether perivascular inflammation initiates, amplifies, or merely reflects advanced arterial disease [174,222]. Taken together, this spatial heterogeneity exposes an important limitation of describing a plaque simply as “inflamed.” Two lesions may contain comparable numbers of macrophages and lymphocytes yet represent profoundly different biological systems: one may be dominated by defective efferocytosis and necrotic-core expansion, another by clonally organized adaptive immunity at a macrophage-rich shoulder, and another by recurrent hemorrhage with hemoglobin-, heme-, and iron-conditioned macrophage populations, while a more fibrotic lesion may retain effective VSMC-mediated matrix repair and cap integrity. The relevant biological unit is therefore not inflammatory-cell burden alone, but the cellular state, its anatomical niche, and the local interactions through which that state becomes consequential. Spatial immune architecture, rather than inflammation considered in aggregate, may ultimately provide the more informative framework for understanding why apparently similar carotid plaques follow different clinical trajectories.

Conventional molecular profiling inevitably compresses the biological complexity of an atherosclerotic plaque. A cytokine concentration or transcript measured in homogenized tissue represents an average across multiple cell populations and anatomically distinct microenvironments, thereby obscuring both the cellular source of the signal and the tissue context in which it was generated. An increase in IL-1β, for example, cannot establish whether inflammatory signaling originated from macrophages bordering the necrotic core, cells associated with neovascular or hemorrhagic regions, or another plaque compartment with a fundamentally different biological significance. The same limitation applies to adaptive immunity: identification of an expanded T-cell clonotype indicates selection but does not reveal whether the corresponding cells are dispersed throughout the lesion or concentrated within the fibrous cap, plaque shoulder, adventitia, or an organized tertiary lymphoid structure. Bulk measurements collapse biologically distinct cellular neighborhoods into a single molecular value and may conceal precisely the heterogeneity that determines plaque behavior [68,155]. Single-cell RNA sequencing has transformed the characterization of human carotid atherosclerosis by resolving previously unrecognized endothelial, myeloid, lymphoid, and VSMC states, but tissue dissociation necessarily separates these cells from the anatomical relationships in which their functions are expressed [223]. Spatial transcriptomic and proteomic approaches address this limitation by reconnecting molecular state with tissue architecture. Recent studies of human carotid endarterectomy specimens have demonstrated that inflammatory macrophage programs, lipid-handling states, neovascularization, VSMC phenotypes, extracellular matrix remodeling, and molecular signatures associated with thin-cap morphology are not uniformly distributed but instead occupy discrete and reproducible plaque territories [224,225]. The next level of biological resolution lies not simply in identifying additional cell states, but in determining where those states reside, which cells surround them, and which structural component of the plaque is exposed to their activity. In this framework, spatial information is not an additional descriptive layer superimposed on single-cell biology; it is necessary for interpreting the biological meaning of the cellular state itself.

## 4. Decoding the Plaque Immune Biography: From Circulating Biomarkers to Single-Cell and Spatial Immunology

Clinical assessment of carotid atherosclerosis is necessarily based on information that can be obtained safely and reproducibly before an intervention. Degree of stenosis, the presence and timing of neurological symptoms, plaque morphology, and increasingly refined ultrasound, CT, and magnetic resonance imaging remain the practical foundations of risk assessment [226,227]. These approaches can identify important structural features of plaque vulnerability, but they were not designed to define the immune state of the arterial wall [228,229,230]. Conversely, much of the mechanistic understanding of human carotid immunobiology has been derived from endarterectomy specimens, obtained only after the lesion has crossed a threshold for clinical intervention. This creates a fundamental translational asymmetry: the greatest biological resolution is available when the plaque has already been removed, whereas the moment at which biological information would be most useful—when treatment decisions are being made—remains dominated by anatomy, symptoms, and imaging. Bridging this gap will require more than identifying another circulating biomarker; it will require determining which features of the plaque immune ecosystem can be inferred reliably before the tissue itself becomes available.

Circulating inflammatory biomarkers represent the most accessible level of such assessment. C-reactive protein, cytokines and chemokines, leukocyte counts, neutrophil-to-lymphocyte ratios, soluble adhesion molecules, and related mediators provide information about systemic inflammatory activity, and several have been associated with carotid plaque burden or imaging features of vulnerability [231,232,233,234]. Their accessibility and suitability for repeated measurement make them attractive for longitudinal studies. Their principal limitation, however, is anatomical ambiguity [235]. Blood integrates inflammatory signals generated throughout the organism; an elevated cytokine concentration cannot establish whether the dominant source is the carotid plaque, another vascular territory, adipose tissue, an intercurrent infection, renal dysfunction, or another inflammatory compartment. Even a biomarker mechanistically implicated in atherosclerosis remains biologically distant from the immune architecture of a particular lesion. Association with plaque vulnerability should not be confused with direct measurement of plaque biology.

Cellular phenotyping moves one step closer to mechanism. Multiparameter flow cytometry and high-dimensional cytometric approaches can resolve circulating monocyte subsets, T-cell differentiation states, regulatory-to-effector relationships, activation and inhibitory receptors, B-cell compartments, and other immune populations, while functional assays can interrogate responses that enumeration alone cannot capture. Yet the same interpretative constraint remains: abundance is not equivalent to state, and state in blood is not necessarily equivalent to state within the plaque. Direct comparison of carotid endarterectomy tissue with peripheral blood has demonstrated substantially greater activation of plaque immune cells and marked differences in inflammatory and prothrombotic gene expression between the two compartments [150]. More recent paired single-cell T-cell receptor (TCR) sequencing has extended this distinction to clonal architecture. Human carotid plaques contain clonally expanded effector T cells that are preferentially enriched within the lesion and display transcriptional evidence of recent TCR engagement; their representation in matched peripheral blood can be substantially different [85]. A plaque-enriched clonotype may therefore constitute only a minor component of the circulating repertoire, whereas a prominent peripheral clone need not be relevant to the carotid lesion. Peripheral blood and plaque should consequently be considered biologically connected but non-equivalent immune compartments, and their discordance may itself contain clinically meaningful information.

Endarterectomy provides access to the complementary side of this relationship. Conventional histopathology remains indispensable because it preserves the architecture in which plaque biology occurs. Fibrous-cap thickness and disruption, necrotic-core dimensions, macrophage infiltration, calcification, neovascularization, intraplaque hemorrhage, and luminal thrombus can be related directly to structural instability and clinical presentation [236,237,238]. Immunohistochemistry adds cellular localization, but its apparent precision should not be overinterpreted. A CD68-positive region identifies neither macrophage ontogeny nor functional state; a CD3-positive infiltrate does not establish antigen specificity or clonality; FOXP3 expression alone does not demonstrate preserved suppressive function; and the presence of neutrophils does not establish NET formation.

Single-cell technologies have changed the second part of that equation. scRNA-seq and complementary single-cell epigenomic approaches have demonstrated that human carotid plaques contain substantially greater cellular heterogeneity than could be inferred from conventional lineage markers. Myeloid cells previously grouped broadly as macrophages resolve into transcriptionally distinct populations associated with inflammatory signaling, lipid handling, tissue adaptation, proliferation, and other functional programs; CD4+ and CD8+ T cells occupy different activation, cytotoxic, memory, and regulatory states; and endothelial cells and VSMCs display multiple phenotypic transitions that challenge conventional definitions of vascular-cell identity [239]. Importantly, these technologies do more than increase the number of cell labels. They change the biological unit of analysis. “Macrophage burden,” for example, becomes less informative once macrophages with potentially divergent functions are recognized within the same lesion, just as total T-cell abundance becomes an incomplete descriptor when only selected populations show clonal expansion and recent antigen-receptor engagement.

Single-cell resolution, however, introduces its own loss of information. Tissue dissociation removes cells from the anatomical relationships in which their functions acquire biological meaning. A macrophage transcriptional state cannot be interpreted fully without knowing whether it lies at the necrotic-core border, within an intraplaque hemorrhage, adjacent to a neovessel, or beneath an intact fibrous cap. Similarly, identification of an expanded T- or B-cell clone does not establish whether that clone occupies the intima, plaque shoulder, adventitia, or an organized tertiary lymphoid structure. Recent spatially resolved studies of human atherosclerotic plaques demonstrate that these distinctions are experimentally accessible, revealing organized cellular neighborhoods and tertiary lymphoid organ-like structures containing expanded and diversified B-cell repertoires [142]. The next advance should therefore not be conceived as another technology added sequentially to histology, cytometry, or single-cell sequencing, but as integration across biological dimensions.

**Figure 5:**
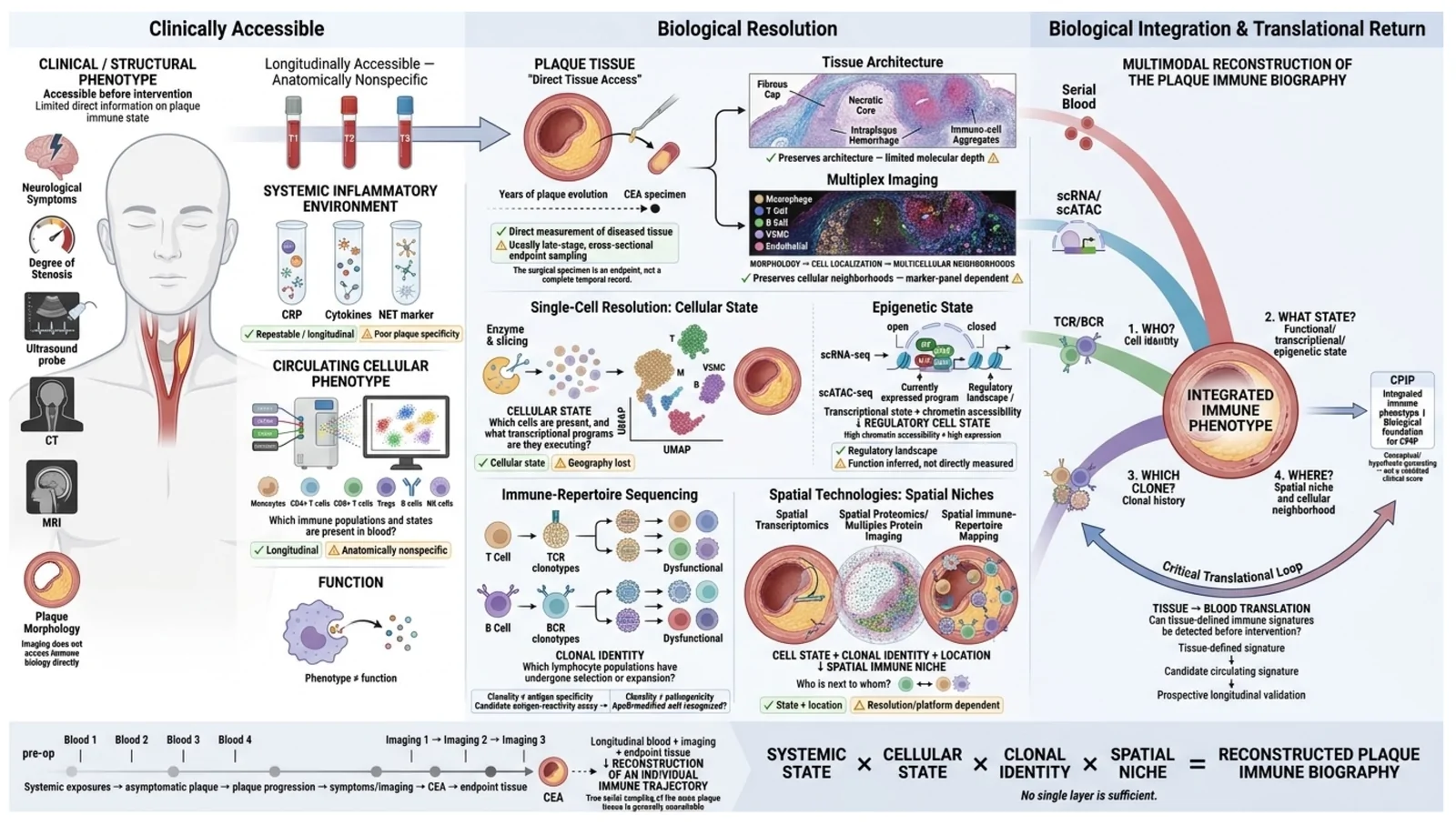
From Blood to Space: Multimodal Reconstruction of the Carotid Plaque Immune Biography. **Abbreviations:** BCR, B-cell receptor; CD4, cluster of differentiation 4; CD8, cluster of differentiation 8; CEA, carotid endarterectomy; CPIP, Carotid Plaque Immune Phenotype; CRP, C-reactive protein; CT, computed tomography; MRI, magnetic resonance imaging; NET, neutrophil extracellular trap; NK, natural killer; scATAC-seq, single-cell assay for transposase-accessible chromatin sequencing; scRNA-seq, single-cell RNA sequencing; TCR, T-cell receptor; Treg, regulatory T cell; UMAP, uniform manifold approximation and projection; VSMC, vascular smooth muscle cell. Created by Urbanowicz T, on 3 October 2026 with www.figurelabs.ai (ID: FL-PUB-20261003-LY2OKA).

The translational objective is ultimately to reconstruct an individual plaque across four linked coordinates: systemic immune state, cellular state, clonal identity, and spatial niche. Blood provides longitudinal accessibility but limited anatomical specificity; endarterectomy provides direct access to the lesion but only at a late and essentially terminal sampling point; single-cell profiling resolves cellular state but disrupts tissue geography; TCR/BCR sequencing identifies clonal selection but does not by itself establish function or anatomical position; and spatial technologies restore the local relationships through which these states and clones become biologically consequential. No individual layer is sufficient. The clinically useful immune phenotype will emerge only when information can be transferred across them and, ultimately, when tissue-defined signatures can be traced back to measurements obtainable before intervention. Figure 5 illustrates this progression from clinically accessible systemic signals to cellular phenotype, clonal architecture, and spatially resolved plaque biology, providing a framework for moving from descriptive plaque immunology toward longitudinal and potentially patient-specific immune phenotyping.

Each analytical level interrogates a different dimension of carotid plaque immunobiology, and none provides a complete representation of the disease in isolation including: peripheral blood and tissue analysis, single-cell transcriptomic and epigenomic approaches, and spatial technologies. The respective strengths and limitations of these approaches are summarized in Table 4. Importantly, their integration should not be viewed as a linear progression toward ever greater technical complexity. Such an approach moves plaque characterization beyond cellular enumeration toward a more functional question: which cells are present, what state they are in, whether they have undergone selective clonal expansion, where they reside, and with which cellular and structural elements they interact.

Single-cell RNA sequencing provides a high-resolution view of the transcriptional state captured at the time of tissue acquisition, but it does not fully explain why a particular state has emerged or which regulatory programs remain available to the cell. This distinction is important because transcription reflects genes that are actively expressed, whereas the underlying chromatin landscape defines a broader repertoire of regulatory elements that are accessible, restricted, or potentially poised for subsequent activation. Single-cell assay for transposase-accessible chromatin sequencing (scATAC-seq) addresses this complementary dimension by mapping open chromatin at single-cell resolution and thereby identifying cell-specific promoters, enhancers, and transcription-factor motif accessibility [247]. Integration of scRNA-seq with scATAC-seq can consequently connect an observed cellular phenotype with the regulatory architecture that supports it, helping to identify transcription-factor networks and regulatory programs that may precede, maintain, or constrain the transcriptional state [248]. This has already proved informative in human carotid atherosclerosis. Combined scRNA-seq and scATAC-seq of endarterectomy specimens identified distinct regulatory landscapes in plaque myeloid and T-cell populations, including differential accessibility of IRF-, STAT-, NFAT-, and KLF-associated motifs [45]. As presented in the aforementioned study, regulatory accessibility and contemporaneous gene expression were not always equivalent: in foam-cell-associated macrophages, for example, increased accessibility of KLF4-related regulatory elements was observed despite relatively low KLF4 transcript abundance. Such discordance is biologically informative rather than contradictory, as it indicates that the transcriptional phenotype reflects only the currently active component of a broader regulatory state. The addition of chromatin accessibility, therefore, shifts single-cell plaque analysis from describing what a cell expresses to understanding the regulatory landscape that permits, stabilizes, or potentially redirects that state.

### 4.1. Arterial-Bed Specificity of Atherosclerosis: Why the Carotid Plaque Is Not a Coronary Plaque

Atherosclerosis is a systemic disease, but its expression is profoundly conditioned by the arterial bed in which it develops. Shared systemic exposures—including dyslipidemia, smoking, diabetes, hypertension, aging, and chronic inflammation—do not produce anatomically or biologically identical lesions throughout the vascular tree. Local geometry, wall structure, mechanical loading, patterns of blood flow, vascular remodeling, and the composition of the surrounding tissue establish distinct microenvironments in carotid, coronary, and peripheral arteries. These differences are relevant to the interpretation of plaque immunobiology because immune-cell recruitment, retention, activation, and spatial organization occur within this local structural context. As many inflammatory pathways are conserved across atherosclerosis, their relative importance and their relationship to plaque progression or clinical events cannot necessarily be extrapolated from one arterial territory to another.

The carotid bifurcation provides a particularly clear example of this principle. Its geometry creates regions of disturbed and oscillatory flow, particularly along the outer wall of the carotid bulb, where low and multidirectional shear stress promotes endothelial dysfunction, altered mechanotransduction, leukocyte adhesion, and lipid retention [249,250]. This spatially restricted hemodynamic environment differs from that encountered within the continuously moving and cyclically compressed coronary circulation and from the mechanical and flow conditions characteristic of larger peripheral arteries [251,252]. Arterial-bed differences begin before an advanced plaque is established: they influence where lesions preferentially develop and subsequently shape the vascular environment within which systemic metabolic and immune signals are translated into local disease [253].

Human pathological studies, particularly the extensive work characterizing coronary atherosclerosis, have further demonstrated that advanced plaques do not progress toward a single stereotyped endpoint. Plaque rupture, superficial erosion, calcified nodules, healed rupture, progressive fibrotic remodeling, and different patterns of calcification represent distinct pathological trajectories rather than interchangeable manifestations of a universal “vulnerable plaque” [254]. This distinction is important when concepts derived from coronary pathology are applied to carotid disease. In the coronary circulation, acute luminal thrombosis following rupture or erosion is directly associated with myocardial ischemia [255]. In carotid atherosclerosis, the clinically relevant pathway may also involve progressive stenosis, plaque-surface disruption with distal embolization, intraplaque hemorrhage, necrotic core expansion, neovascularization, and repeated subclinical remodeling [256]. Consequently, similar degrees of plaque burden or inflammation may have different clinical implications depending on the arterial territory and the structural pathway through which the lesion has evolved.

Intraplaque hemorrhage illustrates this arterial-bed dependence particularly well. In carotid disease, hemorrhage detected by histology or magnetic resonance imaging has emerged as an important feature of high-risk plaque biology and may contribute to rapid plaque expansion by introducing erythrocyte membranes, hemoglobin, heme, iron, and additional lipid into the lesion. These components create a local environment characterized by oxidative and metabolic stress, altered macrophage iron handling, erythrophagocytosis, and inflammatory remodeling [186,257]. Calcification is similarly heterogeneous. Its biological significance cannot be reduced to the amount of calcium present, because microcalcification, larger confluent deposits, calcified nodules, and heavily calcified stable lesions differ in their mechanical context and in their association with plaque progression [258,259]. Accordingly, immune signatures associated with hemorrhage, calcification, or fibrous-cap remodeling should be interpreted in relation to the structural phenotype and arterial territory in which they occur.

These pathological differences also have important implications for the interpretation of experimental atherosclerosis. Animal models reproduce selected components of human disease but differ substantially in vascular anatomy, lesion distribution, plaque maturation, spontaneous rupture, thrombosis, and calcification [260,261]. Findings obtained from the aortic root, brachiocephalic artery, or other commonly studied vascular sites provide essential mechanistic information but do not necessarily recapitulate the temporal and spatial biology of human carotid atherosclerosis [262,263]. The same caution applies to molecular observations derived predominantly from human coronary tissue. Cross-arterial comparisons are valuable precisely because they can distinguish mechanisms that are broadly conserved across atherosclerosis from those whose biological consequences depend on vessel-specific architecture and local pathology.

This distinction becomes particularly important as plaque characterization shifts from conventional histology to single-cell, immune repertoire, and spatially resolved analyses [90]. Identification of an inflammatory macrophage state, a clonally expanded T-cell population, a dysfunctional regulatory program, or a localized inflammasome signature does not, by itself, establish an equivalent contribution to instability across all arterial beds. The biological consequence of a given immune state may depend on whether it is located adjacent to a thin fibrous cap, an endothelial surface undergoing erosion, an intraplaque hemorrhage, a neovascularized region, a calcified interface, or a relatively stable fibrotic compartment. Spatial immune information requires the simultaneous interpretation of the lesion’s structural geography [67].

Seminal human pathological studies by Virmani and colleagues established that advanced atherosclerosis does not converge on a single mode of plaque failure but encompasses distinct substrates, including fibrous-cap rupture, superficial erosion, calcified nodules, healed rupture, and intraplaque hemorrhage [264,265]. This pathological framework is particularly relevant to immune phenotyping because these structural trajectories are unlikely to share an identical cellular or spatial immune architecture. Arterial-bed heterogeneity also extends to peripheral atherosclerosis; direct single-cell comparison of human carotid and femoral plaques has demonstrated differences in leukocyte composition and inflammatory polarization, further supporting the concept that local vascular territory shapes the immune architecture of advanced lesions [239].

The principal anatomical, hemodynamic, pathological, and clinically relevant differences between coronary and carotid atherosclerosis, together with their implications for immune phenotyping, are summarized in Table 5.

For this reason, arterial-bed specificity should be considered an additional interpretative principle of carotid immune phenotyping rather than a limitation that can be resolved simply by increasing molecular resolution. The objective is not to regard carotid atherosclerosis as immunologically independent from systemic atherosclerosis, nor to disregard mechanistic insights derived from coronary and peripheral disease. Rather, shared immune mechanisms should be interpreted within the anatomical, hemodynamic, and pathological context in which they operate. A clinically meaningful carotid immune phenotype must therefore demonstrate that its molecular and cellular features are reproducibly associated with carotid-specific structural phenotypes and, ultimately, with cerebrovascular outcomes. This requirement provides an important boundary condition for the Carotid Plaque Immune Phenotype proposed below: CPIP should not merely catalogue immune features of atherosclerosis, but should determine whether particular combinations of immune history, cellular state, clonal architecture, and spatial organization identify biologically distinct pathways of carotid plaque progression and destabilization.

Additionally, CPIP should not represent a carotid application of a coronary-derived ‘vulnerable plaque’ paradigm; rather, it should test whether combinations of immune history, cellular state, clonality, and spatial organization identify carotid-specific biological pathways leading to embolization, plaque progression, or other clinically relevant cerebrovascular outcomes.

### 4.2. Carotid Plaque Immune Phenotype (CPIP)

The proposed Carotid Plaque Immune Phenotype (CPIP) should be regarded as a conceptual and research framework rather than as a diagnostic or prognostic score. Its novelty does not reside in proposing previously unrecognized immune-cell populations or molecular pathways, many of which are already well established in atherosclerosis, but in organizing these observations into an explicitly multiscale and temporally informed framework for carotid plaque characterization. Existing approaches have largely examined plaque vulnerability through structural morphology, circulating inflammatory biomarkers, cellular abundance, transcriptional states, or individual molecular pathways. CPIP instead proposes that these dimensions be interpreted jointly and in relation to the lesion’s biological history.

Specifically, CPIP extends conventional descriptions of immune heterogeneity by integrating five complementary levels of information: systemic and hematopoietic immune conditioning; cellular and functional immune state; epigenetic or regulatory state; adaptive clonal architecture and antigen experience; and spatial localization within structurally distinct plaque microdomains. The framework distinguishes cellular presence from functional state, functional state from antigen specificity, clonality from pathogenicity, and systemic immune activation from plaque-localized immune activity as presented in Table 6. A further distinction is its explicit incorporation of temporal immune history—the concept that the immune architecture observed at endarterectomy reflects the cumulative effects of prior systemic exposures, hematopoietic conditioning, immune memory, and local tissue remodeling, rather than only the inflammatory state present at the time of plaque removal [11].

Accordingly, CPIP is not proposed as an alternative name for plaque inflammation or vulnerability. Rather, it provides a testable structure for determining whether different combinations of immune history, cellular state, regulatory programming, clonality, and spatial organization define biologically distinct pathways leading to apparently similar structural plaque phenotypes. Its components cannot yet be assigned validated thresholds or numerical weights, and their incremental clinical value beyond established clinical and imaging variables remains unknown. The immediate purpose of CPIP is therefore to generate biologically defined hypotheses that can be evaluated prospectively and reproduced across independent cohorts.

## 5. Limitations of Current Evidence and Critical Gaps in Carotid Plaque Immunology

The increasing resolution with which the immune landscape of atherosclerosis can now be examined should not be confused with an equivalent increase in our ability to establish causality. This distinction is particularly important in human carotid disease. Endarterectomy provides exceptional access to diseased vascular tissue, but the specimen represents a late and highly selected stage of a process that has developed over many years. What is recovered at surgery is therefore a biological endpoint rather than a record of the sequence of events that produced it. A macrophage state, expanded lymphocyte clone, or spatially restricted inflammatory program identified within an advanced plaque may reflect a mechanism that contributed to lesion evolution, a response to an already established lesion, or both. Even very detailed molecular characterization cannot, by itself, resolve this temporal problem.

The source of human carotid tissue also imposes an unavoidable selection. Most molecular studies examine plaques removed because the patient has reached a clinical or anatomical threshold for intervention. These specimens are invaluable for understanding advanced disease. Still, they cannot be assumed to reproduce the biology of earlier lesions or of plaques that remain clinically silent for many years. Comparisons between symptomatic and asymptomatic patients partly address this problem, although symptom status is itself an imperfect biological classifier. A recent cerebral ischemic event may alter circulating and tissue inflammatory responses, while the absence of symptoms does not necessarily identify a stable plaque. The relationship between immune phenotype and clinical presentation is therefore likely to be bidirectional and should be interpreted accordingly.

A second difficulty concerns the distinction between association and biological responsibility. The spatial colocalization of an inflammatory macrophage population with a necrotic core, NETs with regions of endothelial injury, or clonally expanded T cells with a vulnerable plaque compartment is biologically informative, but proximity does not establish causation. The same caution applies to receptor sequencing. TCR or BCR expansion provides evidence that selected lymphocyte populations have undergone expansion or preferential retention. Still, it does not identify the responsible antigen or establish whether the response is protective, pathogenic, or incidental to the vascular process. Likewise, transcriptional states described as inflammatory, regulatory, cytotoxic, exhausted, or resident should not be treated as direct measurements of cellular function without complementary functional evidence. Increasing molecular resolution makes these distinctions more important, not less.

Peripheral blood and plaque tissue introduce a related interpretative problem. Blood permits repeated sampling and thus enables tracking of immune changes over time, but circulating cells provide only an incomplete representation of the immune processes occurring within the arterial wall. The reverse limitation applies to plaque tissue: it provides direct access to the relevant pathological compartment but, in most patients, only at a single time point. A circulating clonotype may be absent from a plaque, while a highly expanded tissue clone may be rare in peripheral blood. Similarly, circulating cytokines, antibodies, or NET-associated markers integrate signals from multiple organs and cannot be assumed to originate from the carotid lesion. Paired blood–plaque analyses are therefore considerably more informative than either compartment alone, but even these remain cross-sectional unless embedded within longitudinal sampling.

Technical resolution also introduces its own sources of uncertainty. Histology, immunohistochemistry, flow cytometry, single-cell RNA sequencing, chromatin accessibility profiling, TCR/BCR sequencing, spatial transcriptomics, and spatial proteomics interrogate different layers of the same biological system and should not be regarded as interchangeable measures of immune activity. Plaque dissociation can selectively lose fragile or matrix-associated populations, while necrosis, calcification, hemorrhage, and fibrosis complicate both tissue processing and spatial sampling. A small tissue fragment may not adequately represent a lesion in which immune composition differs markedly between the fibrous cap, shoulder, necrotic core, neovascularized regions, media, and adventitia. Batch effects, differences in cell annotation, sequencing depth, spatial resolution, and analytical pipelines further complicate comparisons between studies. Some apparent biological heterogeneity may consequently reflect how and where the plaque was sampled as much as differences between patients.

These considerations are particularly relevant to the proposed Carotid Plaque Immune Phenotype (CPIP). CPIP should be regarded as an integrative and hypothesis-generating framework rather than as a validated classification system or clinical risk score. Its purpose is to organize information that is currently considered separately—systemic immune conditioning, cellular composition, functional state, clonal architecture, spatial organization, and structural plaque phenotype—and to provide a framework within which their relationships can be tested. Whether these dimensions form reproducible immune phenotypes across independent populations remains unknown. More importantly, it remains to be established whether such phenotypes provide clinically meaningful information beyond conventional risk factors, degree of stenosis, plaque morphology, and contemporary vascular imaging.

Finally, “plaque vulnerability” itself should not be expected to correspond to a single immune state. Fibrous-cap disruption, intraplaque hemorrhage, necrotic-core expansion, neovascularization, thrombosis, and different patterns of calcification are related but biologically non-equivalent processes. They may share inflammatory mechanisms while retaining distinct cellular and spatial architectures. The objective should therefore not be to identify one universal immune signature of the vulnerable carotid plaque. A more realistic goal is to determine whether reproducible combinations of immune history, cellular state, clonality, and spatial organization define biologically distinct routes to plaque instability.

**Figure 6:**
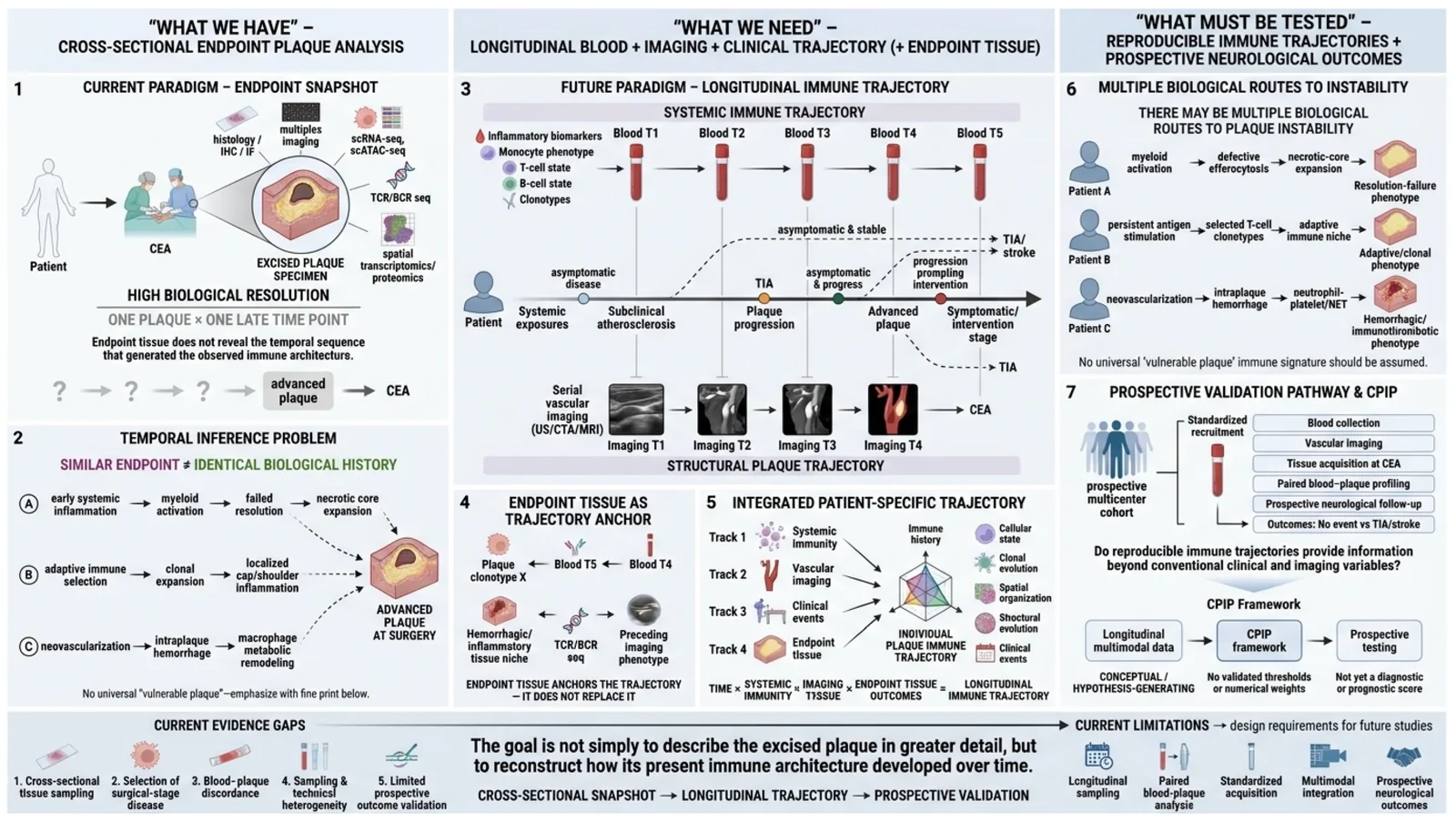
From Cross-Sectional Plaque Analysis to Longitudinal Immune Trajectories in Carotid Atherosclerosis. **Abbreviations:** BCR, B-cell receptor; CEA, carotid endarterectomy; CPIP, Carotid Plaque Immune Phenotype; CTA, computed tomography angiography; IF, immunofluorescence; IHC, immunohistochemistry; MRI, magnetic resonance imaging; scATAC-seq, single-cell assay for transposase-accessible chromatin sequencing; scRNA-seq, single-cell RNA sequencing; TCR, T-cell receptor; TIA, transient ischemic attack; US, ultrasonography. Created by Urbanowicz T, on 3 October 2026 with www.figurelabs.ai (ID: FL-PUB-20261003-Z9YUZO).

A future validation strategy for CPIP should proceed in sequential stages to the proposed framework in Figure 6. First, candidate CPIP domains should be defined using standardized tissue acquisition and multimodal profiling in a discovery cohort, ideally combining paired peripheral blood and endarterectomy tissue with single-cell transcriptomic and/or epigenomic profiling, TCR/BCR repertoire analysis, spatially resolved measurements, conventional histopathology, and preoperative vascular imaging. Second, the resulting immune phenotypes should be compared with established features associated with plaque vulnerability, including fibrous cap disruption or thinning, lipid-rich necrotic core, intraplaque hemorrhage, neovascularization, inflammatory cell infiltration, and clinically validated imaging characteristics. Importantly, CPIP should not be expected to reproduce these structural measures directly; validation should determine whether it identifies biologically distinct immune architectures associated with these phenotypes. Third, reproducibility should be evaluated in independent patient cohorts using prespecified feature definitions, standardized sampling and analytical pipelines, and quantitative assessment of phenotype stability and classification agreement. Where possible, spatial and molecular signatures identified in discovery cohorts should be tested with reduced, technically transferable panels suitable for external validation. Finally, prospective studies should determine whether CPIP-derived phenotypes provide incremental information beyond established clinical variables, stenosis severity, conventional plaque morphology, circulating biomarkers, and contemporary vascular imaging. This could be assessed using multivariable prediction models and measures of discrimination, calibration, and incremental predictive performance for clinically relevant outcomes such as transient ischemic attack, ischemic stroke, or recurrent cerebrovascular events. Such studies would establish whether CPIP represents only a biologically informative taxonomy or can ultimately contribute independent prognostic information.

Demonstrating this will require longitudinal studies, standardized tissue acquisition, paired profiling of peripheral and plaque tissue, integration with high-resolution imaging and histopathology, and, ultimately, prospective association with neurological outcomes. Until such data are available, the immunological biography of the carotid plaque is best viewed as a framework for asking these questions rather than as their final answer.

## 6. Conclusions

The biology of carotid atherosclerosis cannot be fully understood from the composition of the plaque at a single point in time. The lesion encountered during endarterectomy results from a prolonged interaction between systemic exposures and local vascular responses. Disturbed flow and lipid retention establish a permissive environment; metabolic and inflammatory conditions influence hematopoiesis and circulating immune cells; the plaque itself subsequently reshapes recruited leukocytes; and adaptive immune populations may undergo antigen-driven selection, clonal expansion, and functional differentiation. Over time, these processes become embedded in a tissue that is itself undergoing cell death, defective resolution, VSMC remodeling, neovascularization, hemorrhage, and repair.

The emerging picture is not of a uniformly inflamed plaque, but of a mosaic of interacting immune and vascular niches. A clonally expanded T cell in an adventitial lymphoid aggregate, a macrophage failing to clear apoptotic cells at the necrotic-core border, and an activated neutrophil interacting with platelets at the luminal surface all belong to the same diseased artery, but they represent different biological problems. Their significance cannot be inferred from cell number alone. Identity, state, history, interaction, and location must be considered together.

Single-cell, repertoire, and spatial technologies have made this level of interrogation possible in human tissue. The more difficult task now is to determine which elements of this complexity matter clinically and whether they can be recognized before the plaque becomes symptomatic. The field must move from increasingly detailed descriptions of excised lesions toward longitudinal studies that connect systemic immunity, plaque imaging, tissue architecture, and clinical events.

Carotid stenosis describes how much of the lumen has been lost; plaque morphology describes important features of the lesion that remains. Neither, by itself, explains the biological path by which that plaque arrived at its present state. Understanding that path—and determining whether it can be reconstructed in the living patient—may be the next important step in bringing vascular immunology closer to the clinical assessment of carotid disease.

## Figures and Tables

**Table 1:** Systemic and Local Determinants of the Immunological Biography of the Carotid Plaque.

Exposure/Driver	Main Cellular Target	Key Mechanisms	Persistent Biological Effect	Plaque Consequence/Measurable Readout	References
Disturbed or oscillatory flow	Endothelium; recruited leukocytes	Altered mechanotransduction; ↓NO; NF-κB activation; VCAM-1/ICAM-1; chemokine signaling	Sustained endothelial activation at susceptible vascular sites	Leukocyte recruitment and focal inflammation; endothelial markers, vascular imaging, spatial signatures	[69]
Hypercholesterolemia/modified ApoB lipoproteins	Endothelium; macrophages; hematopoietic progenitors; lymphocytes	Lipoprotein retention/modification; scavenger receptors; TLR/NLRP3 signaling; mevalonate pathway	Trained innate immunity, myelopoietic activation and adaptive responses to lipid-associated antigens	Foam-cell formation, inflammation and necrotic-core expansion; lipid/oxLDL measures, immune phenotype, TCR/BCR and single-cell profiling	[70]
Diabetes/chronic hyperglycemia	Endothelium; myeloid cells; neutrophils	AGE–RAGE; oxidative/mitochondrial stress; NF-κB and inflammasome signaling	Metabolic/epigenetic conditioning and persistent endothelial dysfunction	Impaired efferocytosis and enhanced inflammation; HbA1c, AGE markers, myeloid and inflammasome signatures	[71]
Smoking	Endothelium; neutrophils; macrophages; platelets	Combustion-derived particulate and toxicant exposure with direct vascular injury; oxidative stress-dependent and -independent endothelial dysfunction; altered NO signaling; platelet–leukocyte interactions; innate immune activation	Persistent vascular dysfunction and altered innate responsiveness	Oxidative injury and immunothrombosis; exposure markers, endothelial/immune activation and NET-related measures	[72,73]
Obesity/visceral adipose inflammation	Myeloid cells; progenitors; endothelium; perivascular adipose tissue	Adipokines; IL-6/TNF signaling; insulin resistance; altered lipid metabolism	Sustained myeloid and metabolic conditioning	Increased inflammatory tone and leukocyte recruitment; adipokines, monocyte phenotype and metabolic signatures	[74]
Chronic kidney disease	Endothelium; myeloid cells; VSMCs	Uremic toxins; oxidative stress; altered mineral metabolism; chronic inflammatory signaling	Persistent innate and vascular-cell dysfunction	Inflammation, calcification and impaired vascular repair; eGFR, inflammatory/mineral markers and VSMC signatures	[75]
Aging/immunosenescence	Hematopoietic progenitors; myeloid and T cells; endothelium	Inflammaging; mitochondrial dysfunction; senescence; altered immune repertoire	Long-term immune remodeling and reduced immune diversity	Myeloid inflammation and impaired resolution; senescence markers, immune repertoire and single-cell profiling	[76]
Clonal hematopoiesis (CHIP)	Hematopoietic progenitors and myeloid cells	Somatic mutations; altered inflammatory signaling; IL-1β/NLRP3 pathways	Stable production of functionally altered leukocyte clones	Sustained plaque inflammation; CHIP sequencing, clone size and myeloid signatures	[77]
Recurrent infection/microbial exposure	Innate and adaptive immune cells; progenitors; endothelium	PRR/TLR signaling; cytokines; emergency myelopoiesis; selected cross-reactivity	Trained innate responses and adaptive immune memory	Enhanced vascular inflammation; infection history, inflammatory and immune-cell profiles	[78]
Chronic systemic inflammation	Progenitors; circulating leukocytes; endothelium	Persistent cytokine signaling; myelopoiesis; endothelial activation	Repeated innate and adaptive immune conditioning	Sustained leukocyte recruitment and plaque inflammation; cytokines and immune/transcriptomic profiles	[79]
Cell death/DAMP release	Macrophages; dendritic cells; endothelium; VSMCs	HMGB1, ATP and nucleic acids; TLR/purinergic/inflammasome signaling	Recurrent local inflammatory priming	Sterile inflammation and necrotic-core progression; DAMP/inflammasome and spatial signatures	[80]
Defective efferocytosis	Plaque macrophages; apoptotic cells	Impaired apoptotic-cell clearance; altered MerTK signaling; secondary necrosis	Persistent non-resolving inflammation	Necrotic-core expansion and DAMP release; MerTK/efferocytosis markers and spatial macrophage phenotyping	[81]
Intraplaque hemorrhage	Macrophages; endothelium; VSMCs	Hemoglobin/heme; iron handling; oxidative stress; erythrophagocytosis	Local redox and metabolic conditioning	Hemorrhage-associated macrophage niches and plaque destabilization; MRI, iron/heme markers and histology	[82]

**Abbreviations:** AGE, advanced glycation end product; ApoB, apolipoprotein B; BCR, B-cell receptor; CHIP, clonal hematopoiesis of indeterminate potential; DAMP, damage-associated molecular pattern; eGFR, estimated glomerular filtration rate; HMGB1, high-mobility group box 1; ICAM-1, intercellular adhesion molecule 1; NET, neutrophil extracellular trap; NF-κB, nuclear factor kappa B; NLRP3, NOD-, LRR- and pyrin domain-containing protein 3; NO, nitric oxide; oxLDL, oxidized low-density lipoprotein; PRR, pattern-recognition receptor; RAGE, receptor for advanced glycation end products; scATAC-seq, single-cell assay for transposase-accessible chromatin sequencing; scRNA-seq, single-cell RNA sequencing; TCR, T-cell receptor; TLR, Toll-like receptor; VCAM-1, vascular cell adhesion molecule 1; VSMC, vascular smooth muscle cell.

**Table 2:** Adaptive Immune Architecture of Carotid Atherosclerosis: From Cellular Presence to Antigen-Specific Clonal Immunity.

Immune Population/Process	Principal Function and Mechanisms	Clonal or Antigen-Related Features	Potential Role in Plaque Biology	Key Limitation	References
CD4+ Th1 cells	IFN-γ/TNF production; macrophage and endothelial activation	Potential recognition of ApoB and modified lipoproteins; TCR expansion	Sustained inflammation and potential fibrous-cap weakening	Th1 phenotype does not establish plaque-antigen specificity	[131]
Th17/Th1–Th17-like cells	IL-17 signaling; chemokine production and leukocyte recruitment	Potential clonal expansion and phenotypic plasticity	Context-dependent inflammatory amplification	IL-17 may have divergent effects across disease stages	[132]
Regulatory T cells (Tregs)	FOXP3, CTLA-4, IL-10 and TGF-β; suppression of effector responses	Potential antigen-experienced regulatory clones	Restriction of inflammation and maintenance of immune homeostasis	Cell abundance does not establish suppressive function	[95]
Treg plasticity	Loss of regulatory stability; acquisition of Th1/Th17-like characteristics	Shared clonotypes may suggest transitions between functional states	Reduced immune restraint and potentially increased inflammation	Phenotypic overlap does not prove lineage conversion	[132]
CD8+ cytotoxic T cells	Perforin–granzyme, Fas–FasL, IFN-γ and TNF	Clonal expansion; potential plaque-associated antigen recognition	Vascular-cell injury and possible cap destabilization	Cytotoxicity does not establish plaque specificity	[133]
Memory T cells	Rapid recall responses and effector differentiation	Persistent TCR clonotypes across blood and tissue	Potential link between previous antigen exposure and chronic plaque inflammation	Memory does not necessarily imply plaque specificity	[134]
Exhaustion-associated T cells	Co-inhibitory receptors including PD-1, TIM-3, LAG-3 and TIGIT; altered effector and proliferative programs	Expanded populations may reflect persistent antigenic stimulation	Potential restraint of excessive inflammation, but also persistence of functionally altered T-cell states	Inhibitory-receptor expression alone does not establish bona fide T-cell exhaustion	[135]
ApoB-reactive CD4+ T cells	Antigen-specific recognition; regulatory or inflammatory differentiation	ApoB-specific TCR responses	Potential transition from immune regulation to inflammation	Antigen specificity does not determine functional phenotype	[136]
Modified self-reactive T cells	Recognition of oxidized LDL and other modified self-antigens	Antigen-specific responses require functional confirmation	Potential maintenance of chronic adaptive immunity	Recognition of modified self does not establish pathogenic autoimmunity	[131]
TCR clonal expansion	Selective enrichment and persistence of T-cell lineages	TCR sequencing identifies expanded clonotypes	Identification of potentially dominant tissue-enriched immune populations	Clonality does not establish antigen specificity or pathogenicity	[137]
B cells	Antigen presentation, cytokine production and T-cell cooperation	BCR sequencing identifies expanded lineages	Regulatory or inflammatory humoral responses	Total B-cell abundance obscures functional heterogeneity	[138]
Memory B cells	Antigen-experienced responses and rapid reactivation	Persistent BCR lineages	Maintenance of long-term humoral immunity	Memory does not establish plaque-derived antigen recognition	[139]
Plasmablasts/plasma cells	Antibody secretion and potential immune-complex formation	BCR lineages link antibody-secreting and memory populations	Lipid clearance or inflammatory amplification	Antibody secretion does not establish antigen specificity	[140]
Antibodies to modified self-antigens	Recognition of oxLDL and oxidation-specific epitopes	Indirect evidence of antigen-driven B-cell responses	Potential lipid clearance, complement activation or inflammation	Antibody concentration alone does not establish biological function	[141]
BCR clonal expansion	Clonal selection and diversification of B-cell lineages; somatic hypermutation where demonstrated	Expanded and diversified BCR lineages	Potential local humoral immune selection	Clonality does not establish antigen specificity or local germinal-center activity	[142]
Adventitial lymphoid aggregates/TLS	Organized T–B-cell cooperation; antigen presentation; CD40L–CD40 and IL-21 signaling	Potential local clonal selection and B-cell maturation	Maintenance of adaptive immune responses adjacent to the plaque	The degree to which human plaque-associated TLS support sustained local antigen-specific germinal-center reactions and contribute causally to plaque progression remains incompletely established.	[142]

**Abbreviations:** AGE, advanced glycation end product; ApoB, apolipoprotein B; BCR, B-cell receptor; CHIP, clonal hematopoiesis of indeterminate potential; DAMP, damage-associated molecular pattern; eGFR, estimated glomerular filtration rate; HMGB1, high-mobility group box 1; ICAM-1, intercellular adhesion molecule 1; NET, neutrophil extracellular trap; NF-κB, nuclear factor kappa B; NLRP3, NOD-, LRR- and pyrin domain-containing protein 3; NO, nitric oxide; oxLDL, oxidized low-density lipoprotein; PRR, pattern-recognition receptor; RAGE, receptor for advanced glycation end products; scATAC-seq, single-cell assay for transposase-accessible chromatin sequencing; scRNA-seq, single-cell RNA sequencing; TCR, T-cell receptor; TLR, Toll-like receptor; VCAM-1, vascular cell adhesion molecule 1; VSMC, vascular smooth muscle cell.

**Table 3:** Spatial Immune Niches of the Human Carotid Plaque and Their Potential Relationship to Plaque Vulnerability.

Spatial Niche	Predominant Cells	Key Biological Processes/Interactions	Potential Relevance to Plaque Vulnerability	Spatial Characterization	References
Luminal endothelium/blood–plaque interface	Endothelial cells; monocytes; neutrophils; T cells; platelets	Endothelial activation; leukocyte adhesion; platelet–leukocyte interactions; immunothrombosis	Links plaque inflammation with surface thrombogenicity, thrombosis and potential embolization	Multiplex IHC/IF; spatial transcriptomics/proteomics; endothelial-state mapping	[163]
Fibrous cap	VSMCs; macrophages; CD4+/CD8+ T cells	Matrix synthesis/degradation; macrophage proteases; inflammatory signaling; VSMC phenotypic switching	Impaired matrix repair and focal cap weakening may promote surface disruption	Multiplex imaging; matrix imaging; spatial omics; cellular-neighborhood analysis	[164]
Plaque shoulder	Macrophages; T cells; APCs; VSMCs; endothelial cells	Antigen presentation; cytokine signaling; proteolysis; leukocyte recruitment	Convergence of mechanical stress and concentrated immune activity may favor local instability	Spatial transcriptomics; TCR mapping; multiplex IHC/IF	[165]
Necrotic-core border	Lipid-associated/inflammatory macrophages; foam cells; dying macrophages and VSMCs	Lipid and cholesterol-crystal stress; inflammasome activation; defective efferocytosis; secondary necrosis	Failure of clearance promotes necrotic-core expansion and non-resolving inflammation	scRNA-seq with spatial mapping; spatial lipidomics/proteomics; efferocytosis imaging	[68]
Defective-efferocytosis niche	Dysfunctional macrophages; apoptotic/necrotic cells	Impaired MerTK-dependent clearance; secondary necrosis; DAMP release	Promotes non-resolving inflammation and progressive core formation	MerTK/efferocytosis markers; spatial proteomics; macrophage-state profiling	[166]
Inflammasome-active niche	Activated macrophages and other innate immune cells	NLRP3–caspase-1 signaling; IL-1β/IL-18; mitochondrial and lysosomal stress	Sustained local inflammation and impaired tissue repair	NLRP3/caspase-1/IL-1β mapping; spatial omics; single-cell profiling	[167]
Neovascularized region	Microvascular endothelial cells; pericytes; macrophages; lymphocytes	Angiogenesis; microvascular permeability; leukocyte entry; microvessel leakage	Fragile neovessels facilitate inflammation and intraplaque hemorrhage	Microvessel imaging; multiplex IHC/IF; spatial transcriptomics	[168]
Intraplaque hemorrhage niche	Erythrocytes; hemoglobin-handling macrophages; endothelial cells; VSMCs	Erythrophagocytosis; heme/iron metabolism; oxidative stress; cholesterol deposition	Creates a distinct inflammatory–metabolic niche associated with plaque expansion and high-risk morphology	MRI; iron/heme mapping; spatial omics; histology	[169]
Macrophage–VSMC interface	Diverse macrophage and VSMC states	Reciprocal cytokine signaling; matrix remodeling; lipid handling; VSMC phenotypic switching	Balance between inflammatory injury and VSMC-mediated repair may determine stabilization versus progression	scRNA-seq/scATAC-seq; spatial transcriptomics/proteomics	[170]
Neutrophil–platelet/immunothrombotic niche	Neutrophils; platelets; endothelial cells; monocytes; NETs	NETosis; platelet activation; ROS/proteases; coagulation	May connect plaque disruption with acute thrombosis and cerebral embolization	citH3/MPO/NE imaging; platelet markers; multiplex IF; spatial proteomics	[171,172]
Media/intima–media interface	VSMCs; VSMC-derived cells; macrophages	VSMC migration and phenotypic transition; matrix remodeling; calcification	Influences structural remodeling and repair; direct relationship to vulnerability remains uncertain	Spatial transcriptomics; VSMC-state mapping; multiplex imaging	[173]
Adventitial immune compartment	Macrophages; T and B cells; APCs; fibroblasts; vascular/lymphatic cells	Antigen presentation; leukocyte trafficking; stromal–immune signaling	May maintain chronic periarterial innate and adaptive immunity	Spatial omics; multiplex imaging; TCR/BCR sequencing	[97]
Adventitial lymphoid aggregates/TLS-like niches	T cells; B cells; plasma cells; APCs; stromal cells	T–B-cell cooperation; antigen presentation; clonal expansion; potential B-cell maturation	Potential site of local adaptive immune persistence and clonal selection	TCR/BCR sequencing; spatial omics; multiplex histology	[142]
Perivascular adipose tissue/outer vascular niche	Adipocytes; macrophages; lymphocytes; stromal cells	Adipokine/cytokine signaling; oxidative and metabolic stress; outside-in signaling	May amplify or reflect vascular inflammation; directionality remains uncertain	Perivascular tissue profiling; spatial transcriptomics; immune phenotyping	[174]

**Abbreviations:** APC, antigen-presenting cell; ATP, adenosine triphosphate; CCR2, C-C chemokine receptor type 2; citH3, citrullinated histone H3; DAMP, damage-associated molecular pattern; IF, immunofluorescence; IFN-γ, interferon gamma; IHC, immunohistochemistry; IL, interleukin; MPO, myeloperoxidase; MerTK, MER proto-oncogene tyrosine kinase; NE, neutrophil elastase; NET, neutrophil extracellular trap; NLRP3, NOD-, LRR- and pyrin domain-containing protein 3; NO, nitric oxide; ROS, reactive oxygen species; scATAC-seq, single-cell assay for transposase-accessible chromatin sequencing; scRNA-seq, single-cell RNA sequencing; TCR, T-cell receptor; Tfh, T follicular helper cell; TLS, tertiary lymphoid structure; TNF, tumor necrosis factor; VCAM-1, vascular cell adhesion molecule 1; VSMC, vascular smooth muscle cell.

**Table 4:** From Blood to Space: Technologies for Decoding the Carotid Plaque Immune Biography.

Method/Analytical Level	Main Information Obtained	Material	Principal Contribution	Key Limitation	References
Circulating biomarkers	Cytokines, chemokines, soluble inflammatory and endothelial markers	Blood/plasma/serum	Repeatable assessment of the systemic inflammatory environment	Poor plaque and cellular specificity	[234,240]
Peripheral immune phenotyping	Monocyte, T-, B-, NK-cell and Treg/Teff states	Peripheral blood	Longitudinal assessment of circulating immune composition and activation	Blood may not represent plaque-resident immunity	[241]
Functional immune assays	Cytokine production, proliferation, suppression, phagocytosis and antigen responsiveness	Blood-derived or isolated immune cells	Determines whether phenotypically defined cells are functionally active	Requires viable cells; limited spatial information and standardization	[116,242]
Histology/conventional IHC or IF	Plaque morphology and localization of selected cellular or molecular markers	Plaque tissue	Links immune-cell presence to plaque architecture and vulnerability features	Limited molecular depth and usually cross-sectional	[243]
Multiplex tissue imaging	Multiple cellular phenotypes and cell–cell neighborhoods	Plaque sections	Defines multicellular immune niches while preserving tissue architecture	Marker-panel-dependent and hypothesis-driven	[225,244]
scRNA-seq	Cell types, transcriptional states and activation/metabolic programs	Plaque and/or blood cells	Resolves cellular heterogeneity and previously unrecognized immune states	Tissue dissociation removes spatial context; transcript ≠ function	[223]
scATAC-seq/single-cell multi-omics	Chromatin accessibility and regulatory programs integrated with cellular state	Plaque and/or blood cells/nuclei	Identifies regulatory and epigenetic programs potentially reflecting prior immune conditioning	Technically and computationally demanding	[245]
TCR sequencing	T-cell clonality, expansion, diversity and tissue enrichment	Plaque and/or blood T cells	Tracks selected T-cell lineages and plaque-enriched clones	Clonality does not establish antigen specificity or pathogenicity	[133]
BCR sequencing	B-cell clonality, somatic diversification and lineage relationships	Plaque and/or blood B cells	Reconstructs B-cell selection and maturation	Does not establish cognate antigen or antibody function	[142,148]
Paired single-cell + immune-repertoire sequencing	T/B-cell clonotype linked to cellular phenotype	Plaque and/or blood lymphocytes	Connects clonal selection with regulatory, effector, memory or dysfunctional states	Antigen specificity usually remains unresolved	[87]
Antigen-reactivity assays	Direct responses to candidate plaque antigens	Lymphocytes and/or antibodies	Tests whether selected immune responses are genuinely antigen-specific	Candidate-antigen-dependent; unknown antigens may be missed	[87]
Spatial transcriptomics	Gene-expression programs mapped within plaque architecture	Plaque sections	Localizes cellular states to cap, shoulder, core, hemorrhagic and adventitial niches	Platform-dependent resolution; transcript ≠ protein activity	[124]
Spatial proteomics/multiplex protein imaging	Protein-defined cell states and cellular neighborhoods in situ	Plaque tissue	Defines and validates functional cellular neighborhoods	Restricted to selected proteins; technically complex	[155,224]
Spatial immune-repertoire integration	Localization of TCR/BCR clonotypes within plaque niches	Plaque tissue	Connects clonal selection with anatomical position and interacting cells	Technically challenging and not yet standardized	[142,246]
Longitudinal blood–imaging–tissue integration	Evolution of systemic immunity, plaque phenotype and endpoint tissue architecture	Serial blood + imaging + plaque tissue	Reconstructs an individual plaque immune trajectory over time	Requires prospective cohorts; plaque tissue remains endpoint sampling	[241,246]
Integrated longitudinal immune phenotype	Combined systemic, cellular, epigenetic, clonal and spatial information	Multimodal datasets	Proposed endpoint for reconstructing the plaque’s immunological biography and informing CPIP	Conceptual; not yet standardized or clinically validated	[241]

**Abbreviations:** ApoB, apolipoprotein B; ATAC, assay for transposase-accessible chromatin; BCR, B-cell receptor; CPIP, Carotid Plaque Immune Phenotype; IF, immunofluorescence; IHC, immunohistochemistry; NK, natural killer; scATAC-seq, single-cell assay for transposase-accessible chromatin sequencing; scRNA-seq, single-cell RNA sequencing; TCR, T-cell receptor; Teff, effector T cell; Treg, regulatory T cell; VSMC, vascular smooth muscle cell.

**Table 5:** Arterial-Bed Specificity of Atherosclerosis: Comparison of Carotid and Coronary Plaque Biology.

Feature	Coronary Atherosclerosis	Carotid Atherosclerosis	Relevance to Immune Phenotyping
Anatomical and mechanical environment	Epicardial vessels of relatively small caliber; curvature and branch points; continuous cardiac motion, cyclic strain, and compression influence local plaque mechanics.	Large extracranial artery with preferential plaque development at the carotid bifurcation and bulb; marked geometric heterogeneity and less cyclic compression.	Local anatomy modifies endothelial activation, leukocyte recruitment, mechanical stress, and the spatial distribution of inflammatory niches.
Hemodynamic environment	Disturbed flow occurs particularly at branch points and curved segments, superimposed on pulsatile coronary flow.	Low and multidirectional shear stress and oscillatory flow are prominent at susceptible regions of the carotid bifurcation, particularly the outer wall of the bulb.	Hemodynamic context influences endothelial phenotype, adhesion-molecule expression, lipid retention, and localization of inflammatory-cell recruitment.
Dominant advanced plaque phenotypes	Thin-cap fibroatheroma, plaque rupture, plaque erosion, calcified nodules, and healed rupture are well-characterized pathological substrates.	Fibroatheroma, large lipid-rich necrotic core, intraplaque hemorrhage, fibrous-cap disruption, ulceration, calcification, and progressive stenotic remodeling are prominent advanced phenotypes.	A single molecular signature of “vulnerability” is unlikely to represent biologically distinct structural pathways across arterial territories.
Plaque rupture and erosion	Both rupture and superficial erosion are established causes of acute coronary thrombosis; their underlying cellular and extracellular-matrix biology differs.	Surface disruption, rupture, and erosion may promote mural thrombosis and, importantly, distal cerebral embolization.	Immune states associated with cap rupture should not automatically be assumed to characterize erosion or other mechanisms of carotid plaque destabilization.
Intraplaque hemorrhage (IPH)	Occurs in advanced coronary lesions and may contribute to plaque progression and necrotic-core expansion.	Particularly important feature of advanced and high-risk carotid plaques; associated with plaque progression and cerebrovascular risk and accessible to dedicated MRI characterization.	Erythrophagocytosis, hemoglobin/heme exposure, iron handling, oxidative injury, and macrophage responses may generate a distinct local immune microenvironment.
Neovascularization	Adventitial and intraplaque microvessels contribute to inflammatory-cell entry and may facilitate hemorrhage and plaque progression.	Frequently observed in advanced carotid plaques and closely related to IPH and inflammatory infiltration.	Neovascularized regions represent spatially distinct immune niches and should be distinguished from cap, shoulder, necrotic-core, medial, and adventitial compartments.
Calcification	Microcalcification, calcified nodules, and extensive sheet-like calcification represent biologically and mechanically different states.	Calcification is likewise heterogeneous, ranging from small deposits to extensive calcified plaque, often coexisting with fibrosis, necrosis, and hemorrhage.	Calcium burden alone is insufficient for biological classification; morphology and spatial relationship to inflammatory and structural compartments are required.
Vascular remodeling	Positive remodeling may preserve lumen size despite substantial plaque burden; constrictive remodeling can occur in advanced disease.	Plaque enlargement and remodeling coexist with progressive luminal stenosis; morphology and plaque composition may provide information beyond percentage stenosis.	Immune activity should be interpreted against plaque burden and remodeling rather than luminal narrowing alone.
Relationship between plaque and acute event	Local thrombosis after rupture or erosion can acutely obstruct coronary flow, producing myocardial ischemia or infarction.	Clinical events frequently result from plaque-derived thromboembolism to the cerebral circulation, although hemodynamically significant stenosis may also contribute.	The clinically relevant endpoint differs: immune phenotypes should ultimately be validated against arterial-bed-specific mechanisms and outcomes.
Clinically relevant structural imaging	Angiography, intravascular ultrasound, optical coherence tomography, and coronary CT provide complementary information regarding lumen and plaque morphology.	Duplex ultrasonography, CTA, and MRI permit evaluation of stenosis and selected plaque characteristics, including composition, surface morphology, and IPH.	Imaging provides the clinically accessible structural phenotype against which tissue-derived immune signatures could be validated.
Pathological evidence base	Extensive human autopsy and atherectomy studies, including the Virmani pathological framework, have defined rupture, erosion, calcified nodules, healed plaques, and other lesion phenotypes.	Endarterectomy specimens provide exceptionally rich human tissue for histological and molecular characterization but predominantly represent advanced, intervention-selected disease.	Cross-arterial pathology can generate mechanistic hypotheses, but coronary observations require carotid-specific validation.
Major sampling limitation	Human tissue is frequently obtained at autopsy, transplantation, atherectomy, or selected interventional procedures and may not capture longitudinal evolution.	Endarterectomy permits extensive direct tissue analysis but represents a late and essentially terminal sampling point in plaque evolution.	Cross-sectional tissue phenotyping cannot by itself reconstruct the temporal immune trajectory preceding clinical presentation.
Primary clinical endpoints	Acute coronary syndrome, myocardial infarction, chronic coronary syndrome, ischemic cardiomyopathy, and sudden cardiac death.	Transient ischemic attack, ischemic stroke, retinal ischemic events, and recurrent cerebrovascular events.	A clinically meaningful immune phenotype must be validated against territory-specific outcomes rather than a generic definition of cardiovascular risk.
Implication for CPIP	Coronary pathology provides a powerful framework for understanding alternative mechanisms of plaque progression and failure.	Carotid disease requires integration of immune state with bifurcation-specific hemodynamics, IPH, plaque morphology, embolic potential, and cerebrovascular outcomes.	CPIP should represent a carotid-specific biological framework rather than the direct transfer of a generalized or coronary-derived “vulnerable plaque” signature.

**Footnote:** The characteristics shown represent predominant or well-described patterns rather than absolute distinctions between vascular territories. Considerable biological overlap exists between coronary and carotid atherosclerosis. The purpose of the comparison is therefore not to establish mutually exclusive plaque phenotypes, but to emphasize that shared molecular and immune mechanisms operate within arterial-bed-specific anatomical, hemodynamic, structural, and clinical contexts. **Abbreviations:** CPIP, Carotid Plaque Immune Phenotype; CTA, computed tomography angiography; IPH, intraplaque hemorrhage; MRI, magnetic resonance imaging.

**Table 6:** Proposed Carotid Plaque Immune Phenotype (CPIP): An Integrative Framework for Immune Phenotyping of Carotid Atherosclerosis.

CPIP Domain	Central Question	Key Features	Preferred Assessment	Biological Interpretation/Limitation	References
Innate immune activation	Is innate inflammation actively sustained?	Myeloid activation; TLR/NLRP3 signaling; IL-1β/IL-18; DAMP sensing	scRNA-seq; spatial omics; multiplex imaging	Defines active innate signaling; pathway expression does not necessarily prove functional activation	[190]
Macrophage state and resolution	Is macrophage activity inflammatory, reparative or characterized by failed resolution?	Recruited/lipid-associated macrophages; MerTK; efferocytosis; apoptotic/necrotic-cell clearance	scRNA-seq; spatial omics; functional assays	Distinguishes recruitment and activation from failed inflammatory resolution; in situ function remains difficult to establish	[166,187]
T-cell immune state	Is adaptive cellular immunity inflammatory, cytotoxic, regulatory or dysfunctional?	Th1/Th17; CD8+ cytotoxicity; Tregs; Treg/Teff balance; memory/exhaustion programs	scRNA-seq; cytometry; spatial proteomics	Defines functional T-cell architecture; phenotype does not establish antigen specificity	[45]
Adaptive clonality and antigen specificity	Are selected lymphocyte clones expanded, and what do they recognize?	TCR clonotypes; plaque-enriched clones; ApoB/modified-self reactivity	TCR sequencing + scRNA-seq; antigen-reactivity assays	Connects clonal selection with phenotype and antigen recognition; clonality alone does not establish specificity or pathogenicity	[266]
B-cell and humoral immunity	Is humoral immunity clonally selected or locally organized?	B-cell subsets; memory/plasma cells; BCR clonality; somatic hypermutation; antibodies to modified self	BCR sequencing; scRNA-seq; antibody profiling; spatial omics	Defines humoral selection and maturation; antibody levels alone cannot establish origin or biological effect	[142,148]
Neutrophil–platelet immunothrombosis	Is innate immunity linked to thrombogenic activity?	NETs; citH3; MPO; neutrophil elastase; platelet–neutrophil interactions	Multiplex imaging; spatial proteomics	Links inflammation with coagulation and potential plaque-to-thrombus transition; NET markers lack plaque specificity	[171,208]
Endothelial and vascular-cell state	Is the vascular response homeostatic, reparative or maladaptive?	Endothelial activation; VCAM-1/ICAM-1; VSMC phenotypic switching; matrix programs	scRNA-seq/scATAC-seq; spatial omics; histology	Places immune activity within endothelial dysfunction, cap repair and vascular remodeling	[163]
Neovascularization and hemorrhage	Has a hemorrhage-associated inflammatory–metabolic niche developed?	Neovessels; erythrocytes; heme/iron handling; oxidative stress	MRI; histology; spatial omics	Identifies a high-risk microenvironment; temporal relationships cannot usually be reconstructed from endpoint tissue	[169,267]
Spatial immune organization	Where are immune states and selected clones located?	Cellular neighborhoods; cap/shoulder/core/adventitial niches; spatial TCR/BCR clonotypes	Spatial transcriptomics/proteomics; multiplex imaging; spatial repertoire analysis	Converts cellular composition into immune geography; proximity does not prove functional interaction	[124]
Integrated immune trajectory	How did the present plaque immune architecture develop over time?	Serial systemic immunity + imaging + endpoint cellular, clonal and spatial plaque phenotype	Longitudinal blood profiling + vascular imaging + multi-omic plaque analysis	Reconstructs the immunological biography rather than describing only the end-stage lesion; true longitudinal plaque sampling is generally unavailable	[241]

**Abbreviations:** APC, antigen-presenting cell; ApoB, apolipoprotein B; ATP, adenosine triphosphate; BCR, B-cell receptor; citH3, citrullinated histone H3; CPIP, Carotid Plaque Immune Phenotype; CRP, C-reactive protein; CTLA-4, cytotoxic T-lymphocyte-associated protein 4; DAMP, damage-associated molecular pattern; FOXP3, forkhead box P3; ICAM-1, intercellular adhesion molecule 1; IF, immunofluorescence; IHC, immunohistochemistry; IL, interleukin; MerTK, MER proto-oncogene tyrosine kinase; MHC, major histocompatibility complex; MPO, myeloperoxidase; NET, neutrophil extracellular trap; NLRP3, NOD-, LRR- and pyrin domain-containing protein 3; scATAC-seq, single-cell assay for transposase-accessible chromatin sequencing; scRNA-seq, single-cell RNA sequencing; TCR, T-cell receptor; Teff, effector T cell; TGF-β, transforming growth factor beta; Th, T helper; TLR, Toll-like receptor; TLS, tertiary lymphoid structure; Treg, regulatory T cell; VCAM-1, vascular cell adhesion molecule 1; VSMC, vascular smooth muscle cell.

## Data Availability

No new data were created or analyzed in this study. Data sharing is not applicable to this article.

## Abbreviations

AGE	advanced glycation end product
APC	antigen-presenting cell
ApoB	apolipoprotein B
ATAC	assay for transposase-accessible chromatin
ATP	adenosine triphosphate
BCR	B-cell receptor
CCR2	C-C chemokine receptor type 2
CHIP	clonal hematopoiesis of indeterminate potential
citH3	citrullinated histone H3
CPIP	Carotid Plaque Immune Phenotype
CRP	C-reactive protein
CTLA-4	cytotoxic T-lymphocyte-associated protein 4
DAMP	damage-associated molecular pattern
eGFR	estimated glomerular filtration rate
FOXP3	forkhead box P3
HLA-DR	human leukocyte antigen-DR
HMGB1	high-mobility group box 1
ICAM-1	intercellular adhesion molecule 1
IF	immunofluorescence
IFN-γ	interferon gamma
IgM	immunoglobulin M
IHC	immunohistochemistry
IL	interleukin
IPH	intraplaque hemorrhage
KLF4	Krüppel-like factor 4
LAG-3	lymphocyte-activation gene 3
LDL	low-density lipoprotein
MerTK	MER proto-oncogene tyrosine kinase
MHC	major histocompatibility complex
MPO	myeloperoxidase
MRI	magnetic resonance imaging
NE	neutrophil elastase
NET	neutrophil extracellular trap
NF-κB	nuclear factor kappa B
NK	natural killer
NLRP3	NOD-, LRR- and pyrin domain-containing protein 3
NO	nitric oxide
oxLDL	oxidized low-density lipoprotein
PD-1	programmed cell death protein 1
PRR	pattern-recognition receptor
PVAT	perivascular adipose tissue
RAGE	receptor for advanced glycation end products
ROS	reactive oxygen species
scATAC-seq	single-cell assay for transposase-accessible chromatin sequencing
scRNA-seq	single-cell RNA sequencing
TCR	T-cell receptor
Teff	effector T cell
Tfh	T follicular helper cell
TGF-β	transforming growth factor beta
Th	T helper
Th1	T helper 1
Th17	T helper 17
Th9	T helper 9
TIGIT	T-cell immunoreceptor with immunoglobulin and ITIM domains
TIM-3	T-cell immunoglobulin and mucin-domain containing-3
TLR	Toll-like receptor
TLS	tertiary lymphoid structure
TNF	tumor necrosis factor
Treg	regulatory T cell
VCAM-1	vascular cell adhesion molecule 1
VSMC	vascular smooth muscle cell
